# Efficacy and safety of pharmacological and biological therapies for amyotrophic lateral sclerosis: a network meta-analysis

**DOI:** 10.3389/fneur.2026.1754716

**Published:** 2026-04-24

**Authors:** Shixun Zhou, Xinpeng Li, Yurui Jiao, Juan Wu

**Affiliations:** 1 School of Medicine, Nanjing University of Chinese Medicine, Nanjing, China; 2 School of Acupuncture and Tuina, Nanjing University of Chinese Medicine, Nanjing, China; 3 Department of Public Health, School of Medicine, Nanjing University of Chinese Medicine, Nanjing, China

**Keywords:** amyotrophic lateral sclerosis, biological therapies, Lou Gehrig’s disease, motor neuron disease, pharmacological intervention, randomized controlled trial

## Abstract

**Background:**

Amyotrophic lateral sclerosis (ALS) is a progressive neurodegenerative disorder for which disease-modifying treatment options remain limited. This study aimed to systematically assess the efficacy and safety of pharmacological and biological therapies for ALS via a network meta-analysis (NMA).

**Methods:**

PubMed, EMBASE, Cochrane, and Web of Science were searched until February 25, 2025. Randomized controlled trials (RCTs) evaluating any pharmacological or biological intervention in ALS were eligible. Risk of bias was assessed using the Cochrane RoB 2 tool. A Bayesian NMA was performed in R (gemtc package). Effect estimates were expressed as mean differences (MDs) or risk ratios (RRs) with 95% credible intervals (CrIs). Interventions were ranked using the surface under the cumulative ranking curve (SUCRA). Publication bias was explored with funnel plots (Stata 18.0). Subgroup analyses were conducted for drug classes demonstrating significant efficacy and including at least three RCTs.

**Results:**

109 trials involving 16,353 participants were included. The primary outcome was the ALS Functional Rating Scale-Revised (ALSFRS-R); secondary outcomes included forced vital capacity (FVC), mortality, and serious adverse events (SAEs). Compared with placebo, the combination of cell therapy and neuroprotective agents produced the greatest attenuation of ALSFRS-R decline (MD: 3.65, 95% CrI: 1.27–6.05) and was associated with the lowest SAE risk. Receptor agonists ranked highest for preservation of FVC, whereas alkaloids ranked first for mortality reduction; however, no intervention demonstrated a statistically significant survival benefit versus placebo. Within-class subgroup analyses further identified several specific agents, such as masitinib, talampanel, and EH301, as demonstrating relatively consistent efficacy, although substantial heterogeneity remained among enzyme inhibitors.

**Conclusion:**

Cell therapy combined with neuroprotective agents may slow functional decline in ALS. Receptor agonists may help preserve respiratory function. Survival benefits remain inconclusive, underscoring the continued importance of comprehensive supportive care.

**Systematic review registration:**

https://www.crd.york.ac.uk/PROSPERO/view/CRD420251000672, identifier CRD420251000672.

## 1 Introduction

Amyotrophic lateral sclerosis (ALS) is a progressive neurodegenerative disorder characterized by the degeneration of motor neurons in the brain and spinal cord, resulting in progressive weakness and atrophy of the limbs, trunk, neck, and oropharyngeal muscles, often accompanied by pyramidal signs (1). As the most common and severe form of motor neuron disease, ALS has an annual incidence of approximately 2.16 per 100,000 person-years and a median survival of 3–5 years (2, 3). The disease affects both upper and lower motor neurons, and its pathogenesis is believed to arise from a complex interaction between genetic susceptibility and environmental factors (4). Prognosis remains poor, with most patients ultimately dying from respiratory failure secondary to respiratory muscle paralysis within 2–5 years of symptom onset (5).

Pathologically, ALS is marked by selective degeneration of motor pathways and loss of both upper and lower motor neurons. TDP-43 constitutes the principal pathological hallmark, with cytoplasmic inclusions observed in approximately 97% of cases (6). The remaining cases are largely associated with mutations in the *SOD1* or *FUS* genes, causing abnormal cytoplasmic aggregation of the respective proteins (7).

To date, only two agents, riluzole and edaravone, have demonstrated clinical benefit in ALS. Riluzole, approved in 1996, primarily acts through anti-excitotoxic mechanisms, whereas edaravone, approved since 2016 in several Asian countries as well as the United States, Canada, and Switzerland, functions mainly as an antioxidant. However, both confer only modest benefits and do not halt or reverse disease progression (8, 9). Although the number of clinical trials has increased in recent years, positive findings remain limited, underscoring the urgent need for more effective disease-modifying therapies.

Advances in mechanistic research have expanded understanding of ALS pathophysiology, highlighting contributions from oxidative stress, glutamate-mediated excitotoxicity, mitochondrial dysfunction, and pathological protein aggregation (6, 10, 11). Nevertheless, effective treatment options remain limited (12). Given the inability of conventional agents such as riluzole and edaravone to arrest disease progression, several emerging strategies are under active investigation, including stem cell-based, viral vector-mediated, and gene-targeted therapies.

Stem cell-based approaches, using autologous or allogeneic sources (e.g., mesenchymal or neural stem cells), may provide neuroprotection through secretion of neurotrophic factors, modulation of neuroinflammation, and potential replacement or support of damaged neuronal networks, thereby slowing functional decline (13, 14). Viral vector-mediated therapies, most commonly employing recombinant adeno-associated viruses (AAVs), enable efficient central nervous system delivery and sustained therapeutic gene expression, representing a potentially broad platform for both genetic and non-genetic ALS (15, 16). Gene-targeted therapies, such as antisense oligonucleotides (ASOs) or viral vector-mediated gene modulation, aim to delay disease progression by silencing mutant alleles (e.g., *SOD1*, *C9orf72*) or restoring functional protein expression (17, 18). These biologic and genetic approaches represent some of the most promising therapeutic directions in ALS. However, in the absence of direct head-to-head trials, their relative efficacy and safety compared with conventional pharmacologic options remain insufficiently defined.

Most published meta-analyses in ALS have focused on single interventions, limiting cross-treatment comparisons (19, 20). Existing network meta-analyses (NMAs) have primarily examined non-pharmacologic interventions (21), leaving a notable gap in comprehensive comparative evaluation of pharmacologic and emerging biologic therapies. NMA, as an advanced evidence-synthesis method, enables simultaneous comparison of multiple interventions within a unified framework, allowing estimation of relative treatment effects and probabilistic ranking. It also supports comparisons across therapeutic modalities (e.g., conventional drugs versus biologics), thereby offering clinically relevant insights for treatment selection and personalized care.

Therefore, the NMA of biologic and pharmacologic therapies for ALS is crucial to systematically assess their comparative efficacy and safety, optimize therapeutic strategies, and inform both clinical practice and future research directions.

## 2 Materials and methods

Our study followed the Preferred Reporting Items for Systematic Reviews and Meta-Analyses (PRISMA) guidelines and their extensions for NMA (22). The study protocol has been registered on the International Prospective Register of systematic reviews, PROSPERO (2025 CRD420251000672).

### 2.1 Search strategy

The PubMed, Embase, Cochrane Library, and Web of Science databases were systematically searched from inception to February 25, 2025, with language restricted to English. Both Medical Subject Headings (MeSH) terms and free-text keywords were applied, including “amyotrophic lateral sclerosis,” “ALS,” and “randomized controlled trial,” among others. In addition, the reference lists of eligible studies and relevant grey literature were manually screened to identify additional publications. The complete search strategy is provided in Supplementary material 1.

### 2.2 Eligibility criteria

Studies were included if they met the following criteria: (1) Population: Patients diagnosed with ALS; (2) Intervention: Any pharmacological or biological therapy; (3) Comparator: Placebo, riluzole, or edaravone; (4) Study design: Randomized controlled trials (RCTs); (5) Outcomes: The primary outcome was the ALS Functional Rating Scale-Revised (ALSFRS-R). Secondary outcomes included forced vital capacity (FVC), mortality rate, and serious adverse events (SAEs).

Studies were excluded if they: (1) Involved animal or cell experiments, case reports, study protocols, reviews, commentaries, letters, editorials, or conference abstracts; (2) Contained missing, incorrect, or duplicate data; (3) Had unavailable full text; (4) Included overlapping participants; (5) Had a small sample size (*n* ≤ 20).

### 2.3 Study selection and data extraction

All retrieved records were imported into EndNote for reference management. Two reviewers (Shixun Zhou and Xinpeng Li) independently screened the titles and abstracts according to the predefined eligibility criteria, followed by a full-text assessment of potentially eligible studies. Any discrepancies were resolved through discussion or consultation with a third reviewer (Yurui Jiao). Data were extracted independently by two reviewers using a standardized electronic form. The extracted information included: first author, publication year, country, study design, intervention and comparator, sample size, sex, age, treatment duration, and outcome measures.

### 2.4 Quality assessment

Two reviewers (Shixun Zhou and Yurui Jiao) independently assessed the methodological quality of included studies using the Cochrane Risk of Bias 2.0 (ROB 2.0) tool. This tool evaluates five domains: (1) random sequence generation, (2) allocation concealment, (3) blinding, (4) incomplete outcome data, and (5) selective reporting. Each domain was rated as *low risk*, *some concerns*, or *high risk* of bias. A study was considered low risk if all domains were rated as low risk or only one domain was rated as “some concerns.” Studies with any domain rated as *high risk* or with four or more domains rated as *some concerns* were classified as high risk, whereas all others were considered moderate risk. Any discrepancies between reviewers were resolved by consultation with a third reviewer (Juan Wu).

### 2.5 Statistical analysis

Continuous outcomes (ALSFRS-R and FVC) were analyzed using mean difference (MD), while dichotomous outcomes (mortality and SAEs) were analyzed using risk ratios (RRs). A Bayesian NMA model was conducted using the Markov chain Monte Carlo (MCMC) method to estimate the relative efficacy of different treatments. The model was configured with four chains, 10,000 burn-in iterations, and 50,000 simulation iterations, using a thinning interval of 10 and an initial value of 2.5 to ensure convergence. The NMA framework is built upon three fundamental assumptions: transitivity, homogeneity, and consistency. Heterogeneity was assessed using the *mtc.anohe* function from the GeMTC package, with I^2^ < 50% considered acceptable. Inconsistency between direct and indirect evidence was evaluated using the node-splitting method (mtc.nodesplit), where *p*-value >0.05 indicated no significant inconsistency. Convergence was evaluated using the potential scale reduction factor (PSRF), where values between 1 and 1.05 indicated satisfactory convergence. A network diagram was constructed with nodes representing interventions and edges denoting head-to-head comparisons. Cumulative ranking probability plots were generated, and surface under the cumulative ranking curve (SUCRA) values were calculated to estimate the relative ranking probabilities. Publication bias was assessed using funnel plots. All statistical analyses were performed with R 4.5.0 and Stata 18.

## 3 Results

**Figure 1:**
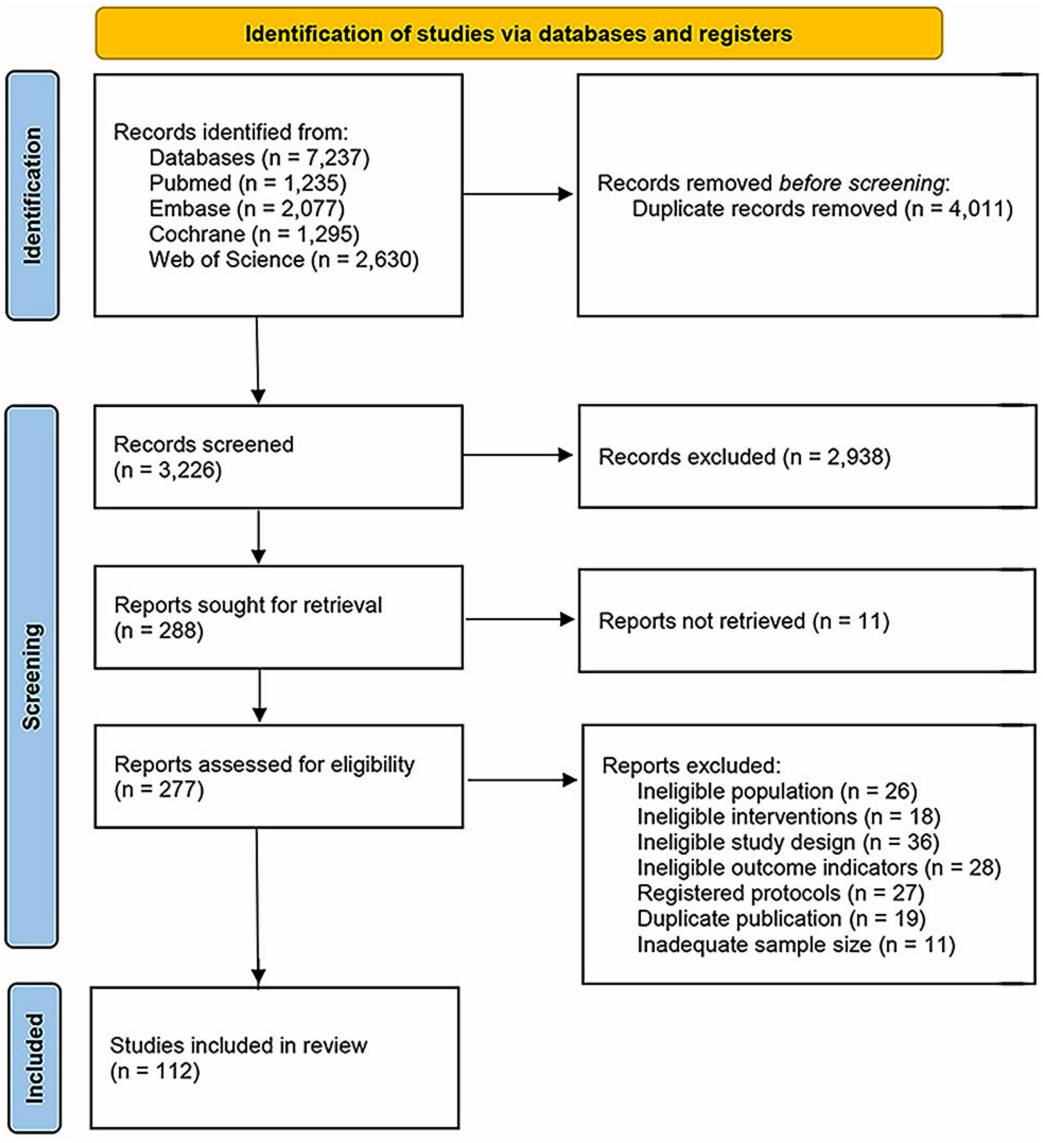
PRISMA 2020 flow diagram of literature search and screening.

7,237 articles were initially identified. After removing 4,011 duplicates, 2,938 were excluded based on title and abstract screening. The full texts of the remaining studies were then reviewed according to the predefined eligibility criteria. Ultimately, 112 articles (23–134) (including 3 errata) met the inclusion criteria. The detailed screening process is illustrated in Figure 1.

### 3.1 Characteristics and quality assessment of included studies

A total of 109 studies were conducted across 19 countries (Australia, Canada, China, France, Germany, India, Iran, Italy, Japan, Korea, Mexico, the Netherlands, Portugal, Spain, the UK, US), involving 16,353 patients (10,252 males and 6,094 females) aged 30–90 years. The basic characteristics of these studies are summarized in Table 1 and detailed in Supplementary Table 1.

**Table 1:** Baseline characteristics of included studies.

Study ID (author, year)	Area	Interventions (vs. Control)	N(I/C)^a^	Gender^b^	Duration	Main outcomes
S. Paganoni 2025 (23)	US	Complement Inhibitor vs. Placebo	122/164	75/115	6 M	ALSFRS-R^A^, MR^B^, AE^C^
M. C. Boll 2025 (24)	México	Mood Stabilizer vs. Placebo	20/18	14/9	18 M	ALSFRS-R, FVC^D^, MR, AE
S. Bhai 2025 (25)	US	Receptor Antagonist vs. Placebo	58/31	31/21	9 M	ALSFRS-R, MR, AE
L. H. van den Berg 2024 (26)	Netherlands	ASO vs. Placebo	79/27	35/13	6 M	ALSFRS-R, MR, AE
S. Pal 2024 (27)	UK	Receptor Antagonist vs. Placebo	183/186	121/127	17 M	ALSFRS-R, MR, AE
J. C. Koch 2024 (28)	Germany	Enzyme Inhibitor vs. Placebo	39/44	23/25	1 M	MR, AE
G. Gianferrari 2024 (29)	Italy	Alkaloid vs. Placebo	36/18	23/13	7.5 M	ALSFRS-R, MR, AE
R. Feng 2024 (30)	China	Microbial Therapeutics vs. Placebo	14/13	8/7	9 M	ALSFRS-R, FVC, MR, AE
M. Benatar 2024 (31)	US	Cell Signaling Modulators vs. Placebo	160/79	106/45	18 M	ALSFRS-R, MR, AE
D. N. Weemering 2023 (32)	Netherlands	Chemically Modified Lipid Therapy vs. Placebo	21/22	10/13	6 M	ALSFRS-R, MR, AE
D. Walk 2023 (33)	US	Dietary Supplements vs. Placebo	14/9	4/5	5 M	ALSFRS-R, MR, AE
S. Vucic 2023 (34)	Australia	Nanomedicine vs. Placebo	23/22	13/13	9 M	ALSFRS-R, FVC, MR, AE
J. Mandrioli 2023 (35)	Italy	Enzyme Inhibitor vs. Placebo	42/21	18/13	4.5 M	ALSFRS-R, MR, AE
M. Liu 2023 (36)	China	Neuroprotective Agent vs. Placebo	93/92	61/70	12 M	ALSFRS-R, FVC, MR, AE
S. Kim 2023 (37)	Korea	Chinese Herbal Medicine vs. Placebo	10/10	5/8	3 M	K-ALSFRS-R, FVC, MR, AE
Angela Genge 2023 (38)	Multinational	Complement Inhibitor vs. Placebo	255/127	161/69	12.5 M	ALSFRS-R, MR, AE
E. Beghi 2023 (39)	Italy	Nanomedicine vs. Placebo	74/73	52/47	6 M	ALSFRS-R, FVC, MR, AE
S. Samadhiya 2022 (40)	India	Free Radical Scavenger+NA^E^ vs. Placebo	15/15	11/9	12 M	ALSFRS-R, MR, AE
R. Oki 2022 (42)	Japan	Dietary Supplements vs. Placebo	65/64	34/40	4 M	ALSFRS-R, FVC, MR, AE
T. M. Miller 2022 (43)	US	ASO vs. Placebo	72/36	43/19	6 M	ALSFRS-R, MR, AE
R. G. Miller 2022 (44)	US	Immunosuppressant vs. Placebo	68/68	45/46	6 M	ALSFRS-R, FVC, MR, AE
M. E. Cudkowicz 2022 (45)	US	Cell Therapy vs. Placebo	95/94	68/59	6 M	ALSFRS-R, MR, AE
H. Aizawa 2022 (46)	Japan	Receptor Antagonist vs. Placebo	22/22	14/15	12 M	ALSFRS-R, MR, AE
M. D. Weiss 2021 (47)	US	Ion Channel Modulators vs. Placebo	14/6	9/5	1 M	MR, AE
B. J. Wainger 2021 (48)	US	Ion Channel Modulators vs. Placebo	23/23	19/13	2.5 M	ALSFRS-R, MR, AE
S. Vucic 2021 (49)	Australia	Immunosuppressant vs. Placebo	72/35	47/23	9 M	ALSFRS-R, FVC, MR, AE

All studies were randomized controlled trials (RCTs).

A, Amyotrophic Lateral Sclerosis Functional Rating Scale – Revised; B, mortality rate; C, adverse events; D, forced vital capacity; E, neuroprotective agent.

^a^Sample size is presented as intervention group vs. control group.

^b^Sex distribution is presented as number of males in intervention group vs. number of males in control group.

**Figure 2:**
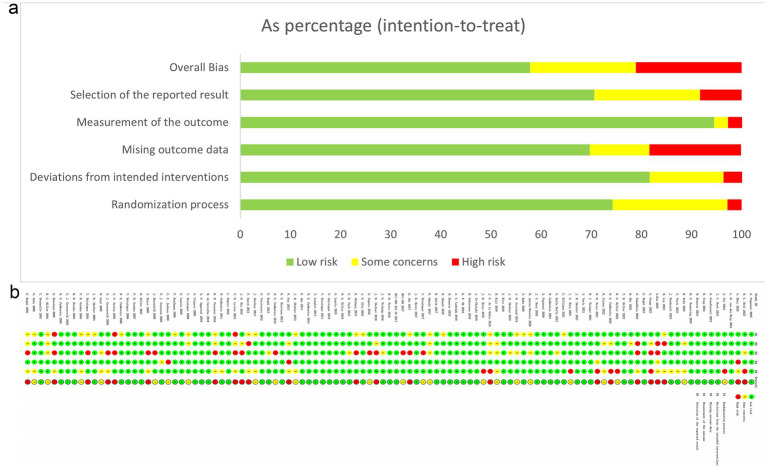
Quality assessment results of included studies.

Most studies were judged to be at low risk of bias. 23 studies exhibited a moderate risk, primarily due to issues such as inadequate blinding, lack of allocation concealment, possible risk identified in the risk-of-bias diagram, or substantial missing data despite appropriate handling. Another 23 studies exhibited high risk owing to baseline imbalances related to the randomization process, deviations from intended interventions, or lack of intention-to-treat analysis. The assessment is detailed in Figure 2.

### 3.2 NMA results

#### 3.2.1 Network graph

**Figure 3:**
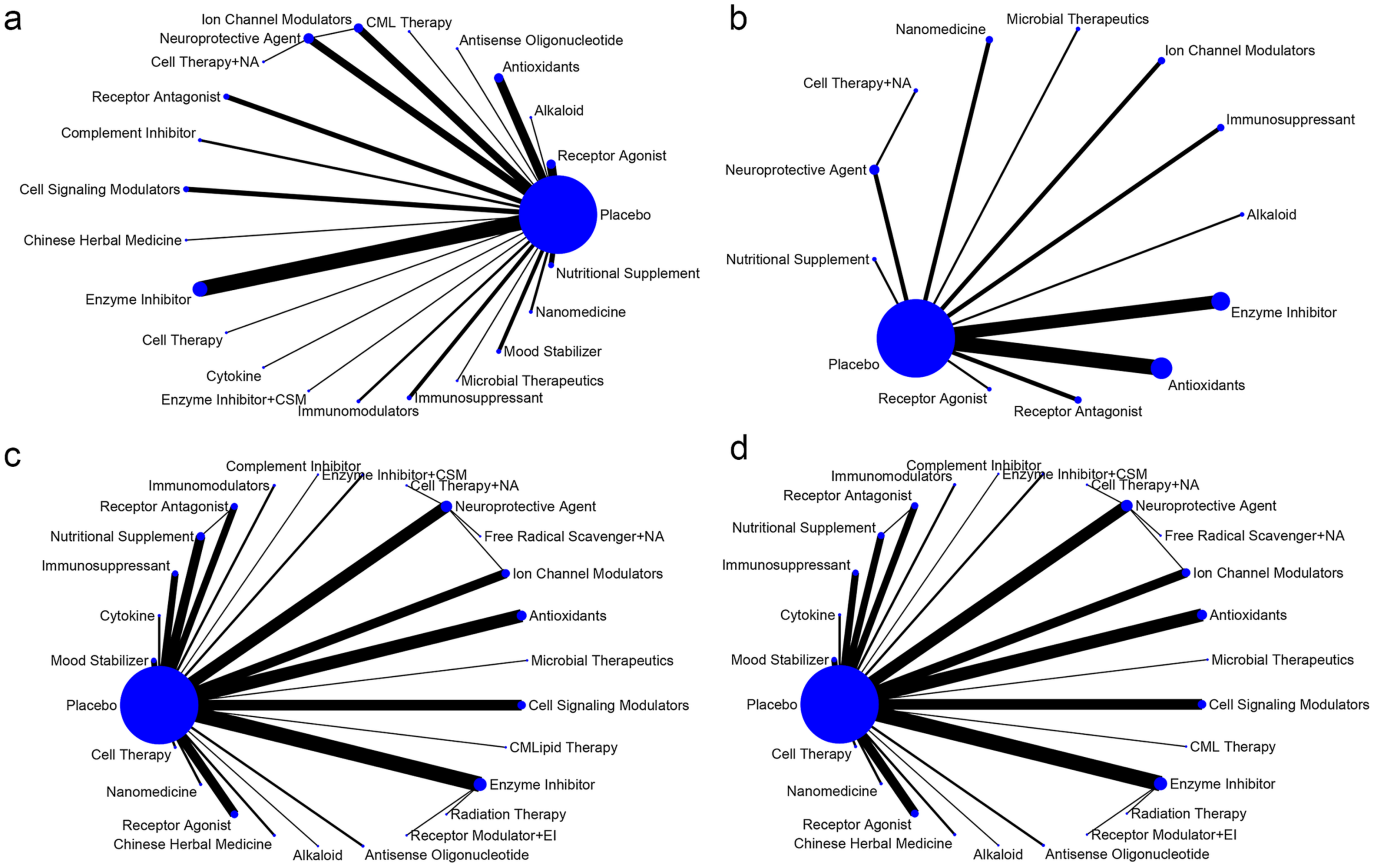
Network plots: **(a)** ALSFRS-R; **(b)** FVC; **(c)** Mortality rate; **(d)** SAEs.

In the network graph, each node represents a specific intervention, with node size proportional to the number of studies involving that intervention-the larger the node, the more studies were included. Edges between nodes indicate direct comparisons between interventions, with edge thickness corresponding to the number of studies for each comparison. Thicker edges indicate a greater number of conducted studies (Figure 3).

Node-splitting analysis was performed to assess loop-specific consistency. Except for FVC, which had no closed loops, *p*-values for ALSFRS-R, mortality rate, and SAEs were all greater than 0.05, indicating no significant local inconsistency. Detailed results are presented in Supplementary material 2, with numeric labels corresponding to the respective interventions.

A Bayesian framework was used to perform the NMA. PSRF values for all outcome models were 1, indicating successful convergence (Supplementary material 3). Differences between the consistency and inconsistency models (ΔDIC) were all below 5, suggesting good model fit and supporting the consistency assumption (Table 2).

**Table 2:** MA model fit results.

Outcome	Consistency model		Inconsistency model		ΔDIC
	DIC	I^2^	DIC	I^2^	
ALSFRS-R	311.89413	34%	315.03378	35%	3.14
FVC	131.52364	40%	131.45155	40%	0.07
Mortality rate	329.2080	0%	333.3683	0%	4.16
Serious adverse events	356.3503	5%	357.7519	4%	1.40

#### 3.2.2 ALSFRS-R

**Figure 4:**
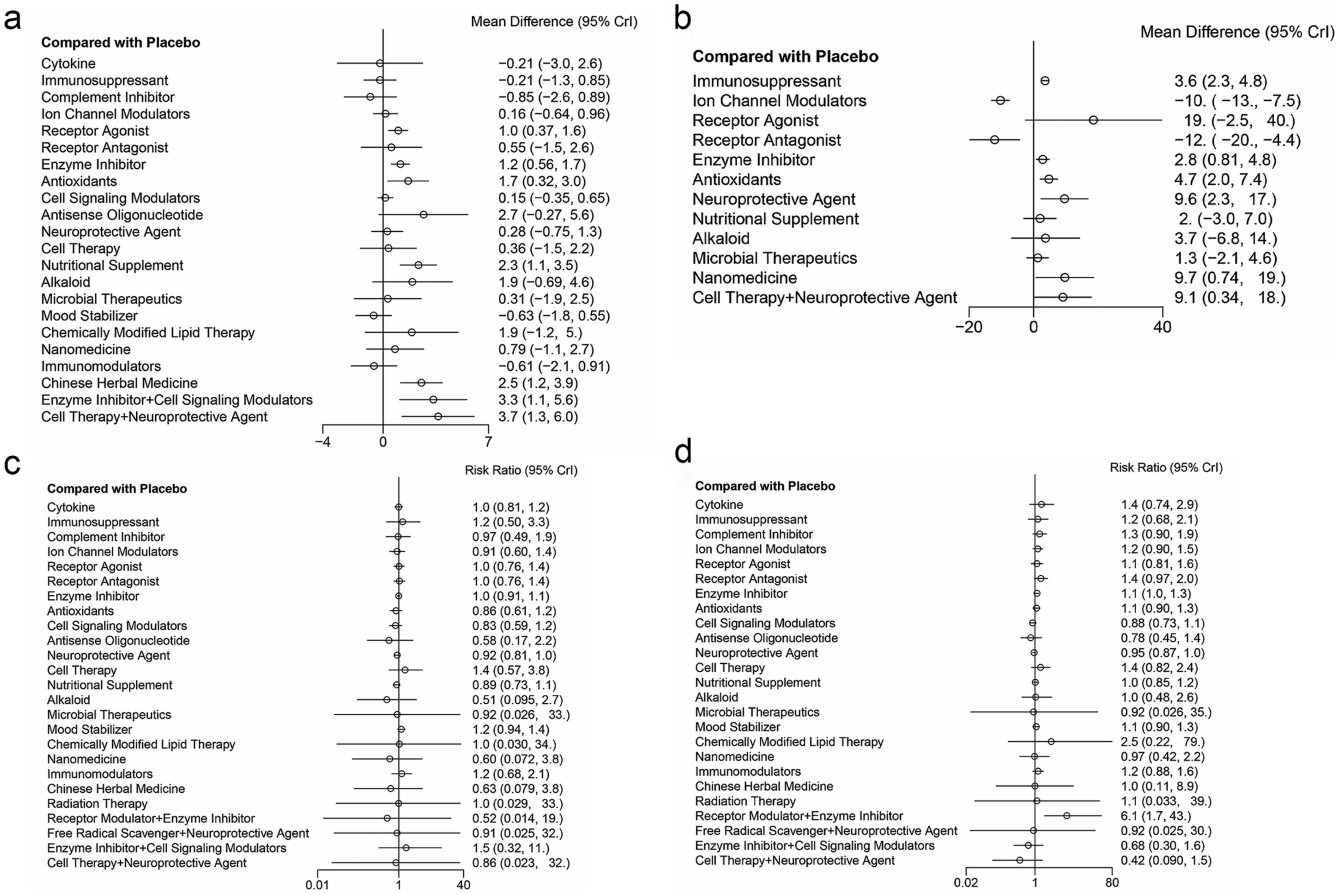
Forest plots: **(a)** ALSFRS-R; **(b)** FVC; **(c)** Mortality rate; **(d)** SAEs.

A total of 72 studies reported ALSFRS-R scores. NMA showed that, compared with placebo, receptor agonist, enzyme inhibitor, antioxidants, nutritional supplement, Chinese herbal medicine, enzyme inhibitor combined with cell signaling modulators, and cell therapy combined with neuroprotective agent significantly improved ALSFRS-R scores (receptor agonist vs. placebo: MD = 1.01, 95% credible intervals (CrI) = 0.38, 1.64; enzyme inhibitor vs. placebo: MD = 1.15, 95% CrI = 0.56, 1.74; antioxidants vs. placebo: MD = 1.66, 95% CrI = 0.32, 3; nutritional supplement vs. placebo: MD = 2.35, 95% CrI = 1.14, 3.55; Chinese herbal medicine vs. placebo: MD = 2.5, 95% CrI = 1.15, 3.89; enzyme inhibitor + cell signaling modulators vs. placebo: MD = 3.32, 95% CrI = 1.08, 5.56; cell therapy + neuroprotective agent vs. placebo: MD = 3.65, 95% CrI = 1.27, 6.05) (Supplementary Table 2; Figure 4).

**Figure 5:**
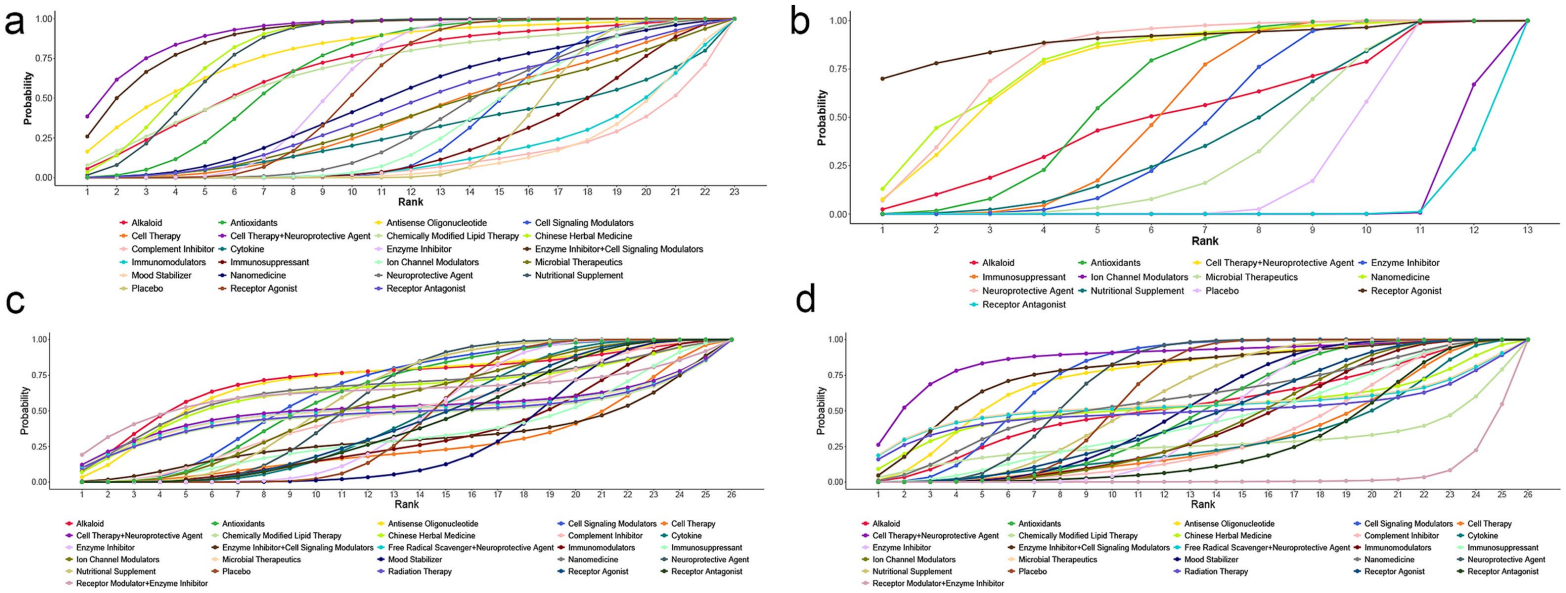
SUCRA probability ranking results. **(a)** ALSFRS-R; **(b)** FVC; **(c)** mortality rate; **(d)** SAEs.

The SUCRA rankings indicated that cell therapy combined with a neuroprotective agent (92.14%) ranked highest, followed by enzyme inhibitor combined with cell signaling modulators (89.85%) and Chinese herbal medicine (83.39%), suggesting that cell therapy with a neuroprotective agent was the most effective intervention in slowing ALSFRS-R decline (Figure 5).

#### 3.2.3 FVC

A total of 29 studies reported FVC. NMA showed that, compared with placebo, ALS patients receiving immunosuppressant, enzyme inhibitor, antioxidants, neuroprotective agent, nanomedicine, or cell therapy combined with neuroprotective agent had significantly higher FVC scores (immunosuppressant vs. placebo: MD = 3.56, 95% CrI = 2.34, 4.77; enzyme inhibitor vs. placebo: MD = 2.82, 95% CrI = 0.83, 4.82; antioxidants vs. placebo: MD = 4.73, 95% CrI = 2.02, 7.44; neuroprotective agent vs. placebo: MD = 9.59, 95% CrI = 2.26, 16.85; nanomedicine vs. placebo: MD = 9.7, 95% CrI = 0.81, 18.53; cell therapy combined with neuroprotective agent vs. placebo: MD = 9.07, 95% CrI = 0.32, 17.77) (Supplementary Table 3; Figure 4).

The SUCRA rankings indicated that receptor agonists (90.14%) ranked highest, followed by neuroprotective agents (81.91%) and nanomedicine (80.25%), suggesting they were the most effective interventions in delaying FVC decline (Figure 5). However, since the comparisons for receptor agonists did not reach statistical significance, these SUCRA rankings should be interpreted with caution.

#### 3.2.4 Mortality rate

A total of 109 studies reported mortality. NMA showed that, compared with placebo, none of the pharmacologic or biologic interventions significantly reduced mortality in ALS patients (Supplementary Table 4; Figure 4).

The SUCRA rankings indicated that alkaloids (72.12%) ranked highest, followed by ASO (70.81%) and nanomedicine (65.26%), suggesting these interventions were the most effective in reducing deaths (Figure 5). However, due to the absence of statistically significant associations, these SUCRA results should be interpreted with caution.

#### 3.2.5 SAEs

A total of 108 studies reported SAEs. NMA showed that, compared with placebo, ALS patients receiving enzyme inhibitor or receptor modulator combined with enzyme inhibitor had significantly higher rates of SAEs (enzyme inhibitor vs. placebo: risk ratio (RR) = 1.12, 95% CrI = 1.01, 1.25; receptor modulator + enzyme inhibitor vs. placebo: RR = 5.91, 95% CrI = 1.69, 39.83) (Supplementary Table 5; Figure 4).

The SUCRA rankings indicated that cell therapy combined with a neuroprotective agent (87.51%) ranked highest, followed by enzyme inhibitors combined with cell signaling modulators (78.21%) and cell signaling modulators alone (75.48%), suggesting these interventions were most effective in reducing SAEs (Figure 5).

### 3.3 Subgroup analyses

Considering that comparisons at the level of specific agents rather than drug classes may yield more granular efficacy information, we conducted subgroup analyses within pharmacological classes that demonstrated significant effects in the primary NMA and included three or more RCTs to explore the relative efficacy of individual agents within each class. The included classes comprised enzyme inhibitors, antioxidants, and receptor agonists (Supplementary material 4). Other significantly effective interventions (e.g., nutritional supplements, traditional Chinese medicine, and combination therapies) were not included in the subgroup analyses due to insufficient study numbers or the inability to form a connected network structure, thereby avoiding inadequate statistical power.

For ALSFRS-R, subgroup analyses successfully identified the most effective specific agents within each class and revealed efficacy differences across molecular targets. Among enzyme inhibitors, masitinib was significantly superior to placebo (MD = 3.72, 95% CrI = 2.74–4.69) and ranked first by SUCRA (91.0%), outperforming other agents in the same class; in contrast, minocycline demonstrated a significant negative effect (Supplementary material 5a). Within the antioxidant class, treatment effects were directionally consistent, with EH301 showing significant benefit and ranking among the top three (MD = 3.0, 95% CrI = 0.06–5.96; SUCRA 63.6%) (Supplementary material 5b). Among receptor agonists, talampanel showed a positive effect and ranked first (SUCRA 97.0%), whereas memantine (MD = −1.39) and perampanel (MD = −4.40) were associated with negative effects, underscoring the importance of receptor subtype selectivity (Supplementary material 5c).

For FVC, subgroup analyses yielded more favorable findings. Among enzyme inhibitors, masitinib remained significantly superior to placebo (MD = 7.50, 95% CrI = 4.69–10.32) and ranked first by SUCRA (93.8%), with a larger effect size than that observed for the ALSFRS-R outcome (Supplementary material 5d). Within the antioxidant class, both EH301 and UDCA demonstrated significant and directionally consistent benefits, with EH301 showing the most pronounced efficacy (MD = 16.39, 95% CrI = 7.22–25.41; SUCRA 98.6%), while UDCA also significantly improved vital capacity (MD = 7.34, 95% CrI = 2.86–11.83). These findings suggest greater within-class consistency for pharmacological interventions targeting respiratory function and provide clearer guidance for clinical decision-making (Supplementary material 5e).

### 3.4 Publication bias

**Figure 6:**
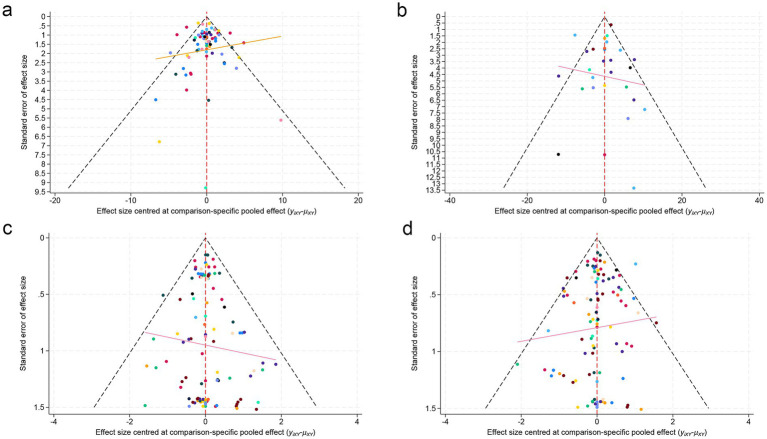
Funnel plots. **(a)** ALSFRS-R; **(b)** FVC; **(c)** Mortality rate; **(d)** SAEs.

Publication bias was examined using comparison-adjusted funnel plots, which appeared symmetrical, indicating the absence of publication bias (Figure 6).

## 4 Discussion

In this NMA of 22 interventions, cell therapy combined with a neuroprotective agent ranked highest in SUCRA for both slowing ALSFRS-R decline and reducing SAEs. Receptor agonists showed the greatest efficacy in preserving FVC, whereas alkaloids ranked highest for reducing mortality risk, although no intervention significantly decreased overall mortality. Notably, cell therapy combined with neuroprotective agents also exhibited potential advantages in mitigating severe adverse reactions. These findings provide a comparative overview of current pharmacologic and biologic interventions in ALS, highlighting promising strategies for both functional preservation and safety management.

These findings are both consistent with and complementary to previous ALS treatment reviews and guidelines (135). Currently, international guidelines continue to recommend riluzole and edaravone as standard therapies. However, our study indicates that certain combination therapies, such as cell therapy combined with neuroprotective agents, may outperform conventional drugs in improving functional outcomes. This discrepancy likely reflects the fact that prior studies predominantly evaluated monotherapies, whereas our NMA incorporates both direct and indirect comparisons, providing a more comprehensive ranking of treatment efficacy (136–138). Furthermore, the modest efficacy observed with conventional oral agents may partly stem from intrinsic challenges in central nervous system drug delivery, including restricted penetration across the blood–brain barrier and insufficient target-specific neuronal uptake (139). This limitation highlights the potential advantages of localized or cell-based delivery approaches, such as cell therapies combined with neuroprotective agents, as identified in our analysis. ALS clinical trials face recruitment challenges and substantial disease heterogeneity, resulting in most intervention studies having sample sizes ≤200 and follow-up periods <18 months, which limits statistical power. In addition, regulatory differences across countries lead to incomplete long-term follow-up data for cell and gene therapies, potentially obscuring their effects on survival (140).

ALSFRS-R and FVC reflect motor neuron and respiratory muscle function (141, 142). Cell therapy combined with neuroprotective agents targets both LMNs and neuromuscular junctions. MSCs, via paracrine effects, release neurotrophic factors (e.g., BDNF, GDNF), angiogenic factors (e.g., VEGF), and anti-inflammatory/anti-apoptotic molecules (e.g., HGF), collectively protecting motor neurons and slowing neuromuscular junction denervation (67, 143). Additionally, MSCs can secrete TGF-β1 and IL-10 to inhibit microglial NLRP3 inflammasome activation, thereby reducing neuroinflammation and abnormal TDP-43 aggregation and deposition in neurons (144, 145). Neuroprotective agents, such as riluzole, inhibit glutamate-mediated excitotoxicity, synergistically mitigating axonal degeneration (146). When combined with MSC therapy, riluzole provides molecular and cellular neuroprotection, while MSCs contribute trophic support and microenvironment modulation, theoretically acting synergistically to delay axonal degeneration and neuronal death. In contrast, ASO therapies such as tofersen represent a precision-medicine paradigm, directly targeting pathogenic *SOD1* mRNA and offering potentially transformative benefits for the small, genetically defined subset of patients with ALS (147). For patients experiencing rapid respiratory decline, receptor agonists targeting neurotrophic factor pathways may selectively enhance survival of phrenic motor neurons via PI3K/Akt and related signaling pathways, offering a potential strategy to slow FVC decline (148).

Despite the observed functional benefits, none of the interventions demonstrated a significant survival advantage. This apparent discrepancy may be attributed to two main factors: First, mortality in end-stage ALS is predominantly caused by respiratory failure, while the relatively short follow-up periods in most clinical trials (typically ≤18 months) may be insufficient to detect delayed survival effects. Second, ALS pathogenesis involves multiple core mechanisms, like TDP-43 proteinopathy and the accumulation of toxic RNA or dipeptide repeat proteins arising from genetic mutations, making it unlikely that single-target interventions can fully counteract the complex, multisystem disruptions in proteostasis and neurotoxicity (6, 149, 150).

Notably, combination therapies (e.g., cell therapy combined with neuroprotective agents) demonstrated superior safety, showing a lower SAE incidence. This advantage may be attributed to the broad immunomodulatory properties of MSCs (143). MSCs regulate T, B, and macrophage cell activity, suppressing peripheral immune-mediated secondary attacks on the CNS, thereby mitigating neuroinflammation-induced tissue damage and treatment-related adverse effects. These findings provide a biological rationale for further investigation of long-term, high-dose combination therapy regimens (151).

Based on current evidence, the combination of cell therapy and neuroprotective agents may represent a promising strategy to slow functional decline in ALS, particularly among patients at an early disease stage or those exhibiting rapid progression (ALSFRS-R decline >1 point/month). For patients with predominantly and rapidly progressive respiratory involvement, the addition of receptor agonists could be considered. However, as cell therapy remains experimental, it should be administered only at qualified tertiary centers under ethical approval, with close monitoring for pulmonary infections and immune-related adverse events. The lack of a confirmed survival benefit further underscores the necessity of comprehensive supportive care, including respiratory support (tracheostomy or permanent assisted ventilation), percutaneous gastrostomy, and nutritional management.

This study represents the largest NMA to date evaluating pharmacological and biological therapies for ALS, encompassing 109 RCTs and 16,353 patients. It comprehensively covered interventions ranging from conventional small-molecule drugs to emerging biologics, including cell and gene therapies. The study adhered strictly to the PRISMA-NMA guidelines and was prospectively registered on the PROSPERO platform, ensuring methodological transparency and reproducibility. Statistical analyses were conducted within a Bayesian framework, which offers advantages in addressing sparse data and generating more robust uncertainty estimates. By calculating SUCRA, our study provided intuitive probabilistic rankings of efficacy and safety, thereby offering clear hierarchical evidence to support clinical decision-making. Furthermore, node-splitting analyses were employed to rigorously assess the consistency across direct and indirect evidence, ensuring the robustness of the pooled results. Importantly, this is the first study to systematically compare the relative efficacy of multiple biologics and conventional pharmacological agents, addressing the long-standing challenge posed by the absence of head-to-head trials and providing high-level evidence to guide individualized therapeutic strategies. Nevertheless, several limitations warrant consideration.

For certain interventions (e.g., cytokines, complement inhibitors, ASOs, cell therapy combined with neuroprotective agents, Chinese herbal medicine, microbial therapeutics, and chemically modified lipid therapy), the number of included studies was limited (fewer than five), and sample sizes were generally small. Consequently, the overall quality of evidence for these interventions was low, primarily due to methodological limitations (risk of bias) and imprecision (wide CrIs). Therefore, the corresponding findings should be interpreted with caution.

For the secondary outcome of FVC, the network failed to form closed loops, precluding the use of node-splitting analyses to assess local inconsistency. Therefore, the FVC-related results relied entirely on indirect comparisons, rendering them inherently less reliable than estimates derived from closed-loop networks with direct evidence. Although statistical heterogeneity was low, variations in patient baseline characteristics, intervention details (e.g., administration routes and dosages), and follow-up durations across studies introduce clinical heterogeneity that may influence the pooled results. Moreover, most included studies had relatively short follow-up periods (≤18 months), leading to an insufficient number of mortality events. This limited the statistical power to detect true differences in survival outcomes and increased the risk of type II errors.

Furthermore, although visual inspection of the funnel plots suggested overall symmetry and did not reveal apparent publication bias, the statistical power of such assessments remains limited. Therefore, the potential impact of unpublished negative results or missing gray literature cannot be completely excluded. In addition, the NMA revealed that, compared with placebo, both enzyme inhibitor monotherapy and receptor modulator-enzyme inhibitor combination therapy significantly increased the risk of SAEs in patients with ALS. Enzyme inhibitor monotherapy resulted in a moderate risk increase (RR = 1.12), possibly due to off-target effects and the additional metabolic burden imposed on patients with already impaired physiological function. This observation aligns with the known safety profiles of several existing ALS therapies (152, 153). In contrast, the risk associated with receptor modulator-enzyme inhibitor combination therapy was markedly higher (RR = 5.91), likely reflecting pharmacodynamic synergism, where interactions between the two drugs within immunological and metabolic pathways lead to toxic effects exceeding those expected from simple additivity (154, 155). Notably, although this combination therapy exhibited a prominent safety signal, the large RR and wide CrI indicate substantial uncertainty, underscoring the need for further studies to validate this finding.

Broad pharmacological classifications may obscure substantial heterogeneity among individual agents. Drugs grouped within the same class often act on distinct molecular targets and can therefore produce markedly different effects, while the categorization of multi-target agents is inherently somewhat subjective. To clarify the contributions of specific drugs within each class, we conducted methodologically rigorous subgroup analyses restricted to categories that showed significant efficacy in the primary analysis and included ≥3 RCTs with a connected network structure. The results demonstrated that although enzyme inhibitors as a class were associated with an increased risk of serious adverse events (SAEs) (RR = 1.12), there was pronounced divergence in efficacy and safety within the class. Masitinib showed the most favorable performance for both ALSFRS-R (MD = 3.72) and FVC (MD = 7.50), ranking first by SUCRA in both outcomes, whereas minocycline exhibited negative effects. These findings not only identify the most effective individual agent but, more importantly, indicate that the overall risk profile of enzyme inhibitors is not uniformly distributed; instead, therapeutic benefit is concentrated in masitinib, suggesting that clinical decision-making should focus on specific agents rather than broad classes. A similar pattern was observed among receptor agonists, where the strong performance of talampanel contrasted with the negative effects of memantine, further underscoring the critical role of molecular target specificity.

Nevertheless, subgroup analyses cannot fully eliminate the residual arbitrariness involved in classifying multi-target drugs, and heterogeneity in baseline patient characteristics and dosing regimens across primary studies limited our ability to examine modifying effects of disease stage and dosage. In addition, several interventions demonstrating favorable efficacy-safety profiles, such as cell therapy combined with neuroprotective agents, could not be subgroup-validated due to the limited number of available RCTs (≤2). Therefore, future research should prioritize multicenter, prospective, large-scale adaptive platform trials with extended follow-up (≥36 months) to evaluate promising but under-studied interventions identified in this analysis, including cell-based combination therapies, selected receptor agonists, and alkaloids. Such trials should place greater emphasis on mortality endpoints to reduce the risk of type II error inherent in short-term studies. In parallel, biomarker-driven precision designs are warranted, incorporating stratification by ALS-FTD-related genotypes (e.g., C9orf72, *SOD1*, TARDBP) or neurofilament light chain (NfL) levels, and leveraging individual patient data network meta-analysis (IPD-NMA) to investigate interactions between treatment effects, disease stage, and genotype (156–158). Moreover, the establishment of internationally harmonized ALS core outcome sets (COS) and standardized adverse event reporting systems is urgently needed to enhance data quality, improve comparability across studies, and strengthen the reliability of future evidence syntheses, including network meta-analyses.

## 5 Conclusion

Based on a comprehensive analysis of ALSFRS-R (motor function), FVC (respiratory function), and safety outcomes, the combination of cell therapy with a neuroprotective agent emerged as the most balanced and promising therapeutic strategy. This regimen ranked highest in delaying motor function decline and reducing SAEs, while also demonstrating significant improvement in FVC compared with placebo.

Riluzole and edaravone, the current standard treatments recommended by international guidelines, were confirmed to exert modest efficacy in slowing functional deterioration. However, their relative performance ranked lower in SUCRA than several novel combination strategies (e.g., the aforementioned cell therapy-based combinations), underscoring the limitations of currently available standard pharmacotherapies. ASO therapies have drawn substantial attention in recent years. In our analysis, ASOs such as tofersen showed potential benefits for selected outcomes, ranking second for mortality reduction, but their effects were primarily confined to patients with defined genetic subtypes (e.g., *SOD1* mutations). This finding supports that ASOs represent a highly effective precision therapy (159, 160). However, for selected genotypes, their applicability remains limited, consistent with recent reports.

A key finding of this study was that none of the evaluated interventions demonstrated a statistically significant survival benefit. This result underscores the therapeutic complexity of ALS: although pharmacological and biologic treatments may slow neurological decline, they remain insufficient to substantially extend overall survival. These results highlight the need for integrating disease-modifying pharmacotherapy with comprehensive supportive care, including respiratory support and nutritional management.

## Data Availability

The original contributions presented in the study are included in the article/Supplementary material, further inquiries can be directed to the corresponding author.
