# Supplementary material for: Efficacy and safety of pharmacological and biological therapies for amyotrophic lateral sclerosis: a network meta-analysis
Source: Front Neurol. 2026 Apr 24;17:1754716. doi: 10.3389/fneur.2026.1754716 (PMC13154608; doi:10.3389/fneur.2026.1754716)
Supplement: Supplementary file 1 [file Table_1.docx]

**Supplementary Table 1:** Baseline characteristics of included studies

| **Authors** | **Year** | **Study Design** | **Area** | **Interventions** | **Sample Size** | **Gender**  **(M/F)** | **Ages** | **Treatment Duration** | **Outcomes** |
| --- | --- | --- | --- | --- | --- | --- | --- | --- | --- |
| S. Paganoni | 2025 | RCT | US | Complement Inhibitor | 122 | 75/47 | 60.1(10.8) | 6M | ALSFRS-R, MR, AE, SVC, NfL |
|  |  |  |  | Placebo | 164 | 115/49 | 57.8(11.3) | 6M |  |
| M. C. Boll | 2025 | RCT | México | Mood Stabilizer | 20 | 14/6 | 53.6(8.7) | 18M | ALSFRS-R, MR, FVC, BMI, AE |
|  |  |  |  | Placebo | 18 | 9/9 | 51.2(6.8) | 18M |  |
| S. Bhai | 2025 | RCT | US | Receptor Antagonist | 58 | 31/27 | 62.3(11.0) | 9M | ALSFRS-R,NfL, TNF-α, ALS-CBS, NPI-Q, MR, AE |
|  |  |  |  | Placebo | 31 | 21/10 | 63.2(12.1) | 9M |  |
| L. H. van den Berg | 2024 | RCT | Netherlands | Antisense Oligonucleotide | 79 | 35/46 | 60.0(4.9) | 6M | AE, MR, ALSFRS-R, HHD, serum pharmacokinetics parameters, SVC |
|  |  |  |  | Placebo | 27 | 13/14 | 58.4(9.0) | 6M |  |
| S. Pal | 2024 | RCT | UK | Receptor Antagonist | 183 | 121/62 | 62.8(9.9) | 17M | ALSFRS-R, MR, AE |
|  |  |  |  | Placebo | 186 | 127/59 | 60.7(11.1) | 17M |  |
| J. C. Koch | 2024 | RCT | Germany | Enzyme Inhibitor | 39 | 23/16 | 59.0(10.0) | 1M | AE, Safety, Tolerability, MR |
|  |  |  |  | Placebo | 44 | 25/19 | 61.0(8.4) | 1M |  |
| G. Gianferrari | 2024 | RCT | Italy | Alkaloid | 36 | 23/13 | 56.6(12.6) | 7.5M | ALSFRS-R, AE, Tracheostomy-free, MR |
|  |  |  |  | Placebo | 18 | 13/5 | 57.3(9.4) | 7.5M |  |
| R. Feng | 2024 | RCT | China | Microbial Therapeutics | 14 | 8/6 | 50.9(9.5) | 9M | ALSFRS-R, FVC, MMSE, HAMD, HAMA, MR, AE |
|  |  |  |  | Placebo | 13 | 7/6 | 49.3(14.7) | 9M |  |
| M. Benatar | 2024 | RCT | US | Cell Signaling Modulators | 160 | 106/54 | 58.0(11.3) | 18M | CAFS, ALSFRS-R, NfL, SVC, MR, AE |
|  |  |  |  | Placebo | 79 | 45/34 | 56.6(10.0) | 18M |  |
| D. N. Weemering | 2023 | RCT | Netherlands | Chemically Modified Lipid Therapy | 21 | 10/11 | 62.9(8.3) | 6M | AE, MR, ALSFRS-R, SVC, NfL |
|  |  |  |  | Placebo | 22 | 13/9 | 58.7(9.2) | 6M |  |
| D. Walk | 2023 | RCT | US | Dietary Supplements | 14 | 4/10 | 60.0(9.9) | 5M | AE, MR, ALSFRS-R |
|  |  |  |  | Placebo | 9 | 5/4 | 56.4(10.0) | 5M |  |
| S. Vucic | 2023 | RCT | Australia | Nanomedicine | 23 | 13/10 | 57.0(13.3) | 9M | MR, ALSFRS-R, AE, FVC, ALSSQOL-SF |
|  |  |  |  | Placebo | 22 | 13/9 | 61.3(10.9) | 9M |  |
| J. Mandrioli | 2023 | RCT | Italy | Enzyme Inhibitor | 42 | 18/24 | 56.0(11.9) | 4.5M | Treg cells, ALSFRS-R, NfL, ALSAQ40, MR, AE |
|  |  |  |  | Placebo | 21 | 13/8 | 55.6(12.9) | 4.5M |  |
| M. Liu | 2023 | RCT | China | Neuroprotective Agent | 93 | 61/32 | 54.9(10.4) | 12M | ALSFRS-R, FVC, CGI,  Total MRC score, MR, AE |
|  |  |  |  | Placebo | 92 | 70/22 | 52.8(10.2) | 12M |  |
| S. Kim | 2023 | RCT | Korea | Chinese Herbal Medicine | 10 | 5/5 | 54.9(12.3) | 3M | K-ALSFRS-R, MRC score, FVC, VAS pain, MR, AE |
|  |  |  |  | Placebo | 10 | 8/2 | 57.1(10.0) | 3M |  |
| Angela Genge | 2023 | RCT | multinational | Complement Inhibitor | 255 | 161/94 | 58.6(10.6) | 12.5M | ALSFRS-R, CAFS, SVC, Time to ventilation assistance-free MR, AE |
|  |  |  |  | Placebo | 127 | 69/58 | 58.0(11.0) | 12.5M |  |
| E. Beghi | 2023 | RCT | Italy | Nanomedicine | 74 | 52/22 | 59.3(10.4) | 6M | FVC, FEV1, ALSFRS-R, ALSAQ-40 (Eating and drinking), MR, AE |
|  |  |  |  | Placebo | 73 | 47/26 | 56.0(10.0) | 6M |  |
| S. Samadhiya | 2022 | RCT | India | Free Radical Scavenger+Neuroprotective Agent | 15 | 11/4 | 52.8(6.9) | 12M | mRS, ALSFRS-R, ALSFRS, Japanese ALS severity score, serum creatinine, MR, AE |
|  |  |  |  | riluzole | 15 | 9/6 | 54.2(3.6) | 12M |  |
| R. Oki | 2022 | RCT | Japan | Dietary Supplements | 65 | 34/31 | 61.2(11.4) | 4M | ALSFRS-R, AE, FVC, MR, ALSAQ-40 |
|  |  |  |  | Placebo | 64 | 40/24 | 60.8(12.1) | 4M |  |
| T. M. Miller | 2022 | RCT | US | Antisense Oligonucleotide | 72 | 43/29 | 48.1(12.6) | 6M | ALSFRS-R, Total SOD1 Concentration in CSF, Concentration of NfL in Plasma, AE, MR |
|  |  |  |  | Placebo | 36 | 19/17 | 51.2(11.6) | 6M |  |
| R. G. Miller | 2022 | RCT | US | Immunosuppressant | 68 | 45/23 | 57.8(10.9) | 6M | ALSFRS-R, FVC, MR, AE |
|  |  |  |  | Placebo | 68 | 46/22 | 56.6(10.8) | 6M |  |
| M. E. Cudkowicz | 2022 | RCT | US | Cell Therapy | 95 | 68/27 | 48.1(9.7) | 6M | ALSFRS-R, MR, AE, Safety(TEAEs) |
|  |  |  |  | Placebo | 94 | 59/35 | 49.1(8.4) | 6M |  |
| H. Aizawa | 2022 | RCT | Japan | Receptor Antagonist | 22 | 14/8 | 61.6(9.8) | 12M | ALSFRS-R, MR, MMT, AE |
|  |  |  |  | Placebo | 22 | 15/7 | 62.6(9.4) | 12M |  |
| M. D. Weiss | 2021 | RCT | US | Ion Channel Modulators | 14 | 9/5 | 58.5(10.8) | 1M | RMT, AE, RMT, MR, **CSP**, peak CMAP |
|  |  |  |  | Placebo | 6 | 5/1 | 52.0(11.3) | 1M |  |
| B. J. Wainger | 2021 | RCT | US | Ion Channel Modulators | 23 | 19/4 | 58.8(9.5) | 2.5M | SICI, SDTC, RMT, MR, AE, ALSFRS-R, SVC |
|  |  |  |  | Placebo | 23 | 13/10 | 57.4(8.0) | 2.5M |  |
| S. Vucic | 2021 | RCT | Australia | Immunosuppressant | 72 | 47/25 | 60.1(9.8) | 9M | ALSFRS-R, MRC, FVC, SNIP(cmH2O), MR, AE |
|  |  |  |  | Placebo | 35 | 23/12 | 58.7(11.0) | 9M |  |
| J. M. Shefner | 2021 | RCT | US | Cell Signaling Modulators | 117 | 67/50 | 60.1(11.0) | 3M | FVC, ALSFRS-R, MR, AE, muscle strength mega-score |
|  |  |  |  | Placebo | 115 | 68/47 | 59.6(10.6) | 3M |  |
| J. S. Mora | 2021 | RCT | Spain | Enzyme Inhibitor | 130 | 83/47 | 55.5 (10.6) | 9.5M | MR, ALSFRS-R, AE |
|  |  |  |  | Placebo | 133 | 80/53 | 55.2(10.6) | 9.5M |  |
| C. Milligan | 2021 | RCT | US | Immunosuppressant | 14 | 11/3 | 61.9(8.7) | 4M | MR, CSF, plasma, AE |
|  |  |  |  | Placebo | 8 | 5/3 | 59.2(8.6) | 4M |  |
| E. Dalla Bella | 2021 | RCT | Italy | Receptor Agonist | 50 | 29/21 | 60.0(10.0) | 6M | ALS Milano-Torino staging, MR, ALSFRS-R, SVC, AE |
|  |  |  |  | Placebo | 49 | 29/20 | 61.0(12.0) | 6M |  |
| M. Cudkowicz | 2021 | RCT | US | Ion Channel Modulators | 329 | 207/122 | 59.0(11.2) | 12M | SVC,ALSFRS-R, MR, AE |
|  |  |  |  | Placebo | 167 | 100/67 | 59.7(10.8) | 12M |  |
| S. Paganoni | 2020 | RCT | US | Enzyme Inhibitor+Cell Signaling Modulators | 87 | 61/26 | 57.6(10.4) | 6M | ALSFRS-R, Plasma pNF‑H, SVC, MR, AE |
|  |  |  |  | Placebo | 48 | 32/16 | 57.3(7.6) | 6M |  |
| J. S. Mora | 2020 | RCT | multinational | Enzyme Inhibitor | 130 | 83/47 | 55.5(10.6) | 12M | ALSFRS-R, ALSAQ-40, FVC, overall MR, AE |
|  |  |  |  | Placebo | 133 | 80/53 | 55.2(10.6) | 12M |  |
| R. Juntas-Morales | 2020 | RCT | France | Neuroprotective Agent | 20 | 14/6 | 58.9(4.8) | 6M | MR, AE, SVC, ALSFRS-R, Maximal Inspiratory Pressure |
|  |  |  |  | Placebo | 10 | 7/3 | 59.6(5.8) | 6M |  |
| S. Babu | 2020 | RCT | multinational | Dietary Supplements | 22 | 16/6 | 55.5(11.3) | 9M | ALSFRS-R, ATLIS, SVC,  AE, MR, HHD |
|  |  |  |  | Receptor Antagonist | 21 | 17/4 | 60.3(10.6) | 9M |  |
| J. M. Statland | 2019 | RCT | US | Enzyme Inhibitor | 60 | 40/20 | 58.4(10.2) | 12M | ALSFRS-R, FVC, MR, ALSQOL, AE |
|  |  |  |  | Placebo | 20 | 13/7 | 57.5(8.5) | 12M |  |
| J. M. Shefner | 2019 | RCT | multinational | Cell Signaling Modulators | 373 | 263/110 | 56.8(10.0) | 12M | Supine SVC, HHD, AE, MR, ALSFRS-R |
|  |  |  |  | Placebo | 188 | 123/65 | 55.9(10.6) | 12M |  |
| Nilo Riva | 2019 | RCT | Italy | Receptor Agonist | 29 | 18/11 | 58.4(10.6) | 1.5M | MAS, NRS, MRC, AE , MR, ALSFRS-R, FVC |
|  |  |  |  | Placebo | 30 | 16/14 | 57.2(13.8) | 1.5M |  |
| R. Kaji | 2019 | RCT | Japan | Dietary Supplements | 123 | 71/52 | 62.4(9.6) | 42M | ALSFRS-R, AE, MR |
|  |  |  |  | Placebo | 123 | 71/52 | 62.2(10.7) | 42M |  |
| J. E. de la Rubia | 2019 | RCT | Spanish | Antioxidants | 13 | 9/4 | 56.9(9.1) | 4M | ALSFRS-R, MRC, FVC, EMG, MR, AE |
|  |  |  |  | Placebo | 14 | 8/6 | 55.6(10.5) | 4M |  |
| J. D. Berry | 2019 | RCT | US | Cell Therapy | 36 | 25/11 | 50.3(11.9) | 6M | AE, MR, CSF, ALSFRS-R |
|  |  |  |  | Placebo | 12 | 10/2 | 53.5(9.1) | 6M |  |
| A. Al-Chalabi | 2019 | RCT | multinational | Ion Channel Modulators | 66 | 47/19 | 56.5(8.3) | 1M | sitting SVC, supine SVC, ALSFRS-R, MR, AE |
|  |  |  |  | Placebo |  |  |  | 1M |  |
| B. Oskarsson | 2018 | RCT | US | Ion Channel Modulators | 23 | 15/8 | 62.4(12.5) | 1M | Number of Cramps, AE, Average Muscle Cramp, MR |
|  |  |  |  | Placebo |  |  |  | 1M |  |
| K. W. Oh | 2018 | RCT | Korea | Cell Therapy+  Neuroprotective Agent | 32 | 18/14 | 53.7(7.7) | 6M | ALSFRS-R, AALS, CSF, AE, MR, FVC |
|  |  |  |  | Neuroprotective Agent | 27 | 11/16 | 52.5(9.4) | 6M |  |
| A. C. Ludolph | 2018 | RCT | German | Enzyme Inhibitor | 126 | 68/58 | 60.1(11.2) | 12M | MR, ALSFRS-R, SVC, SEIQoL, AE |
|  |  |  |  | Placebo | 125 | 84/41 | 60.4(10.2) | 12M |  |
| Michael Benatar | 2018 | RCT | US | Cell Signaling Modulators | 17 | 6/11 | 51.1(12.3) | 12M | AE, suirvival, ALSFRS-R, MR, FEV6 slope, CAFS |
|  |  |  |  | Placebo | 19 | 7/12 | 50.8(10.3) | 12M |  |
| M. Ahmadi | 2018 | RCT | Iran | Antioxidants | 27 | 21/6 | 51.5(13.1) | 12M | MR, ALSFRS-R, MMT, CMAPamplitude, AE |
|  |  |  |  | Placebo | 27 | 18/9 | 58.5(9.6) | 12M |  |
| R. Smith | 2017 | RCT | US | Antioxidants | 31 | 34/26 | 57.8(11.1) | 1M | CNS-BFS, ALSFRS-R, AE, MR, AE |
|  |  |  |  | Placebo | 29 |  |  | 1M |  |
| V. Meininger | 2017 | RCT | multinational | Neuroprotective Agent | 152 | 103/49 | 55.7(10.4) | 11.5M | ALSFRS-R, AE, MR |
|  |  |  |  | Placebo | 151 | 97/54 | 55.5(11.0) | 11.5M |  |
| J. D. Berry | 2017 | RCT | US | Immunosuppressant | 18 | 10/8 | 56.5(8.0) | 1M | AE, FEV1, ALSFRS-R, Total lymphocyte counts, MR, SVC |
|  |  |  |  | Placebo | 10 | 5/5 | 55.2(11.3) | 1M |  |
| K. Abe | 2017 | RCT | Japan | Antioxidants | 52 | 31/21 | 55.3(10.8) | 6M | ALSFRS-R, FVC, MR, AE, Modified Norris scale, ALSAQ-40 |
|  |  |  |  | Placebo | 44 | 28/16 | 57.4(10.7) | 6M |  |
| K. Odachi | 2017 | RCT | Japan | Receptor Antagonist | 10 | 3/7 | 71.6(10.4) | 3M | Pain VAS score, ALSFRS, MR, AE |
|  |  |  |  | Placebo |  |  |  | 3M |  |
| THE WRITING GROUP | 2017 | RCT | Japan | Antioxidants | 13 | 7/6 | 57.6(6.9) | 6M | ALSFRS-R, FVC, MR, Modified Norris Scale, ALSAQ-40, AE |
|  |  |  |  | Placebo | 12 | 6/6 | 62.0(9.5) | 6M |  |
| ALS 19 Study Group | 2017 | RCT | Japan | Antioxidants | 69 | 38/31 | 60.5(10.0) | 12M | ALSFRS-R,FVC, MR, Modified Norris Scale, ALSAQ-40, AE |
|  |  |  |  | Placebo | 68 | 41/27 | 60.1(10.0) | 12M |  |
| M. D. Weiss | 2016 | RCT | US | Ion Channel Modulators | 19 | 12/7 | 58.0(10.0) | 3M | ALSFRS-R, SVC, MR, AE, Muscle cramp frequency, cramps |
|  |  |  |  | Placebo | 20 | 10/10 | 57.0(7.0) | 3M |  |
| J. G. Weikamp | 2016 | RCT | Netherlands | Enzyme Inhibitor | 10 | 2/8 | 63.7(9.4) | 1M | Drool Rating Scale, MR, AE, VAS:diurnal degreeof saliva |
|  |  |  |  | Radiation Therapy | 10 | 5/5 | 60.4(9.0) | 1M |  |
| J. M. Shefner | 2016 | RCT | multinational | Cell Signaling Modulators | 178 | 131/47 | 56.1(11.7) | 3M | ALSFRS-R, SVC, MR, AE, HHD, MVV, SNIP |
|  |  |  |  | Placebo | 210 | 148/62 | 56.8(10.6) | 3M |  |
| E. Nagata | 2016 | RCT | Japan | Receptor Agonist | 29 | 21/8 | 59.7(9.0) | 3.5M | ALSFRS-R, Grip strength, ALSAQ40 communication, ALSAQ40 eating and drinking, MR, AE |
|  |  |  |  | Placebo | 7 | 6/1 | 58.7(10.1) | 3.5M |  |
| A. E. Elia | 2016 | RCT | Italy | Antioxidants | 15 | 10/5 | 54.0(12.2) | 13.5M | ALSFRS-R, MR, FVC, MRC scale, AE |
|  |  |  |  | Placebo | 14 | 9/5 | 58.2(12.9) | 13.5M |  |
| K. Shibuya | 2015 | RCT | Japan | Ion Channel Modulators | 30 | 20/10 | 66.2(9.9) | 6M | ALSFRS-R, SDTC, MrC, MR, FVC, AE |
|  |  |  |  | Neuroprotective Agent | 30 | 16/14 | 67.4(7.2) | 6M |  |
| S. B. Park | 2015 | RCT | Australia | Ion Channel Modulators | 26 | 16/10 | 54.2(9.8) | 8M | ALSFRS-R, MR, AE, Neurophysiological Index, CMAP |
|  |  |  |  | Placebo | 28 | 16/12 | 53.5(10.6) | 8M |  |
| R. G. Miller | 2015 | RCT | US | Immunosuppressant | 45 | 14/31 | 43.0(9.6) | 6M | ALSFRS-R, responders, AE, Inflammatory biomarkers  (IL)-18, LPS, MR |
|  |  |  |  | Placebo | 42 | 13/29 | 41.0(9.8) | 6M |  |
| G. Lauria | 2015 | RCT | Italy | Receptor Agonist | 103 | 55/48 | 59.4(9.7) | 12M | MR, Overall events, AE,  ALSFRS-R, ALSAQ-40, SVC |
|  |  |  |  | Placebo | 97 | 50/47 | 58.6(10.5) | 12M |  |
| N. Amirzagar | 2015 | RCT | Iran | Cytokine | 20 | 13/7 | 51.3(8.6) | 3M | ALSFRS-R, ALSAQ-40, MMT, CMAP, MR, AE |
|  |  |  |  | Placebo | 20 | 12/8 | 52.5(11.6) | 3M |  |
| V. Meininger | 2014 | RCT | multinational | Neuroprotective Agent | 57 | 44/13 | 56.8(9.5) | single dose | AE, SVC, MMT, MR, ALSFRS-R, Ozanezumab pharmacokinetic parameters |
|  |  |  |  | Placebo | 19 | 13/6 | 54.7(11.4) | single dose |  |
| T. Lenglet | 2014 | RCT | multinational | Neuroprotective Agent | 259 | 167/92 | 57.3(11.2) | 6.5M | MR, ALSFRS-R, BMI, MMT, SVC, AE |
|  |  |  |  | Placebo | 253 | 164/89 | 55.7(11.2) | 6.5M |  |
| M. E. Cudkowicz | 2014 | RCT | US,Canada | Enzyme Inhibitor | 340 | 209/131 | 56.0(10.0) | 14.2M | MR, ALSFRS-R, VC, HHD,  AE |
|  |  |  |  | Placebo | 173 | 101/72 | 55.0(10.0) | 12.3M |  |
| K. Abe | 2014 | RCT | Japan | Antioxidants | 101 | 63/38 | 57.7(9.4) | 6M | ALSFRS-R, FVC, Grip, Pinch strength, ALSAQ40, MR, AE |
|  |  |  |  | Placebo | 104 | 69/35 | 57.2(8.8) | 6M |  |
| J. M. Shefner | 2013 | RCT | multinational | Cell Signaling Modulators | 36 | 18/18 | 57.0(12.2) | 0.5M | AE, MR, AE, ALSFRS-R, MVV |
|  |  |  |  | Placebo | 13 | 7/6 | 53.0(12.4) | 0.5M |  |
| Weidong Pan | 2013 | RCT | China | Chinese Herbal Medicine | 23 | 14/9 | 51.6(7.2) | 6M | ALSFRS, SF-36physical function(PF), MR, AE |
|  |  |  |  | Placebo | 19 | 11/8 | 50.1(4.2) | 6M |  |
| K. E. Morrison | 2013 | RCT | UK | Mood Stabilizer | 107 | 71/36 | 59.7(9.9) | 18M | MR, AE, ALSFRS-R, HADS, EuroQoL, AE |
|  |  |  |  | Placebo | 107 | 77/30 | 59.5(11.5) | 18M |  |
| M. E. Cudkowicz | 2013 | RCT | multinational | Neuroprotective Agent | 474 | 307/167 | 56.8(11.3) | 12M | CAFS, ALSFRS-R, MR, SVC,HHD, AE |
|  |  |  |  | Placebo | 468 | 298/170 | 57.3(11.3) | 12M |  |
| E. Beghi | 2013 | RCT | Italy | Neuroprotective Agent | 42 | 24/18 | 61.0(8.3) | 12M | loss of independence, AE, MR, ALSFRS-R, FVC, MRC |
|  |  |  |  | Placebo | 40 | 14/26 | 63.0(7.9) | 12M |  |
| E. Verstraete | 2012 | RCT | Netherlands | Mood Stabilizer | 66 | 37/19 | 59.5(11.0) | 10M | MR, ALSFRS-R, FVC, AE |
|  |  |  |  | Placebo | 67 | 43/24 | 59.0(11.7) | 10M |  |
| J. Shefner | 2012 | RCT | US | Cell Signaling Modulators | 67 | 46/21 | 58.1(10.5) | 6~7D | AE, MR, MVV, Submaximal handgrip endurance, Pulmonary function tests |
|  |  |  |  | Placebo |  |  |  |  |  |
| F. Saccà | 2012 | RCT | Italy | Receptor Agonist | 20 | 14/6 | 63.7(8.5) | 12M | NAA(Cre + Cho), IGFBP-3, ALSFRS-R, HOMA-IR, MR, AE |
|  |  |  |  | Placebo | 20 | 10/10 | 61.7(8.3) | 12M |  |
| J. H. Min | 2012 | RCT | Korea | Antioxidants | 25 | 15/10 | 50.1(2.2) | 4M | MR, ALSFRS-R, FVC,  AE |
|  |  |  |  | Placebo | 28 | 20/8 | 48.3(1.7) | 4M |  |
| T. D. Levine | 2012 | RCT | US | Receptor Agonist | 9 | 15/12 | 58.0* | 6M | ALSFRS-R, tau, pNF-H, MR, AE |
|  |  |  |  | Tretinoin | 18 |  |  | 6M |  |
| L. Dupuis | 2012 | RCT | Germany | Receptor Agonist | 109 | 63/46 | 58.9(10.6) | 13M | MR, ALSFRS-R, EQ-5D, NIV, AE |
|  |  |  |  | Placebo | 109 | 71/38 | 59(10.4) | 13M |  |
| M. Cudkowicz | 2011 | RCT | US | Neuroprotective Agent | 26 | 19/7 | 58.2(11.0) | 3M | ALSFRS-R, MR, VC, AE |
|  |  |  |  | Placebo | 27 | 14/13 | 55.8(9.0) | 3M |  |
| R. M. Pascuzzi | 2010 | RCT | US | Receptor Antagonist | 40 | 28/12 | 56.3(11.5) | 9M | TQNE arm strength megaslope, ALSFRS, MR, AE |
|  |  |  |  | Placebo | 19 | 12/7 | 52.6(7.0) | 9M |  |
| M. de Carvalho | 2010 | RCT | Portugal | Receptor Antagonist | 32 | 21/11 | 58.9(9.6) | 12M | ALSFRS, FVC, MRC, MUNE, SF-36, AE, MR |
|  |  |  |  | Placebo | 31 | 21/10 | 58.3(10.0) | 12M |  |
| S. P. Aggarwal | 2010 | RCT | US,Canada | Mood Stabilizer | 40 | 30/10 | 58.3(10.2) | 5.4M | MR, ALSFRS-R, SVC, ALSSQOL, AE |
|  |  |  |  | Placebo | 44 | 24/20 | 55.5(11.9) | 5.4M |  |
| S. Piepers | 2009 | RCT | Netherlands | Mood Stabilizer | 82 | 56/26 | 57.0(10.1) | 16M | MR, ALSFRS, AE,  Sequential MR analysis |
|  |  |  |  | Placebo | 81 | 52/29 | 57.4(8.9) | 16M |  |
| V. Meininger | 2009 | RCT | multinational | Immunomodulators | 184 | 115/69 | 55.7(11.7) | 12M | ALSFRS-R, MR, MMT, SVC, SF-36, AE |
|  |  |  |  | Placebo | 182 | 110/72 | 56.7(12.1) | 12M |  |
| G. Lauria | 2009 | RCT | Italy | Receptor Agonist | 12 | 7/5 | 53.0(8.6) | 24M | MR, AE, ALSFRS-R, FVC |
|  |  |  |  | Placebo | 11 | 6/5 | 58.0(10.6) | 24M |  |
| P. Kaufmann | 2009 | RCT | US | Antioxidants | 75 | 40/35 | 56.5(10.8) | 9M | ALSFRS-R, FVC, FSS, AE, SF-36–PCS(physical), MR, SF-36–MCS(mental) |
|  |  |  |  | Placebo | 75 | 46/29 | 57.4(11.0) | 9M |  |
| C. E. Jackson | 2009 | RCT | US | Enzyme Inhibitor | 11 | 4/7 | 67.0(6.8) | 8M | ALSFRS-R, MR, AE, SEIQOL-DW |
|  |  |  |  | Placebo | 9 | 6/3 | 64.0(11.9) | 8M |  |
| E. J. Sorenson | 2008 | RCT | US | Cytokine | 167 | 110/57 | 53.9* | 24M | MMT, AUC, MR, AE, ALSFRS-R, |
|  |  |  |  | Placebo | 163 | 100/63 | 54.8* | 24M |  |
| J. Rosenfeld | 2008 | RCT | US | Dietary Supplements | 53 | 35/18 | 56.0(10.0) | 9M | MVIC, MR, ALSFRS, FVC, AE |
|  |  |  |  | Placebo | 54 | 27/27 | 59.0(11.0) | 9M |  |
| T. Meyer | 2008 | RCT | Germany | Immunosuppressant | 18 | 10/8 | 56.0(14.0) | 3M | Mean heart rate, FVC, AE,  ALS-FRS-R, MMTCS, MR |
|  |  |  |  | Riluzole | 19 | 13/6 | 56.0(10.0) | 4M |  |
| R. Miller | 2007 | RCT | multinational | Cell Signaling Modulators | 110 | 68/42 | 53.5(12.1) | 6~11M | ALSFRS-R, MR, FVC, MMT, AE |
|  |  |  |  | Placebo | 108 | 69/39 | 55.5(11.5) | 6~11M |  |
| P. H. Gordon | 2007 | RCT | US | Enzyme Inhibitor | 206 | 143/63 | 58.6(11.8) | 9M | ALSFRS-R, FVC, MMT, AE, MR |
|  |  |  |  | Placebo | 206 | 131/75 | 57.7(10.9) | 9M |  |
| V. Meininger | 2006 | RCT | multinational | Enzyme Inhibitor | 199 | 116/83 | 57.1(11.7) | 12M | MR, ALSFRS-R, MMT, VC, AE |
|  |  |  |  | Placebo | 201 | 139/62 | 56.7(12.1) | 12M |  |
| M. E. Cudkowicz | 2006 | RCT | US | Enzyme Inhibitor | 201 | 129/72 | 54.5(11.8) | 12M | MVIC, ALSFRS-R, VC, MUNE, MR, AE |
|  |  |  |  | Placebo | 99 | 66/33 | 55.0(12.4) | 12M |  |
| S. N. Scelsa | 2005 | RCT | US | Enzyme Inhibitor | 23 | 18/5 | 47.9* | 9M | ALSFRS, MMTCS, FVC,  SF-36, AE, MR |
|  |  |  |  | Placebo | 23 | 15/8 | 49.5* | 9M |  |
| G. J. Groeneveld | 2005 | RCT | Netherlands | Dietary Supplements | 88 | 57/31 | 57.1(11.2) | 10M | AE, MR, renal function |
|  |  |  |  | Placebo | 87 | 63/24 | 58.4(10.9) | 10M |  |
| M. Graf | 2005 | RCT | Germany | Dietary Supplements | 83 | 54/29 | 59.0(11.0) | 18M | MR, Norris limb, AE |
|  |  |  |  | Placebo | 77 | 50/27 | 57.0(11.0) | 18M |  |
| J. M. Shefner | 2004 | RCT | US | Dietary Supplements | 50 | 33/17 | 59.0(12.5) | 6M | MVIC, ALSFRS, FVC, MUNE, MR, AE |
|  |  |  |  | Placebo | 54 | 31/23 | 59.0(10.8) | 6M |  |
| V. Meininger | 2004 | RCT | multinational | Neuroprotective Agent | 581 | 361/220 | 55.8(11.6) | 18M | MR, tracheostomy, PAV, VC, ALSFRS, AE |
|  |  |  |  | Placebo | 286 | 168/118 | 55.2(11.8) | 18M |  |
| P. H. Gordon | 2004 | RCT | US | Enzyme Inhibitor | 10 | 7/3 | 54.0(12.4) | 6M | AE, MR, Tolerability, MMT, ALSFRS-R, FVC, MR, |
|  |  |  |  | Placebo | 9 | 4/5 | 58.6(12.7) | 6M |  |
| B. R. Brooks | 2004 | RCT | US | Receptor Modulator+  Enzyme Inhibitor | 65 | 42/23 | 54.8(12.8) | 1M | CNS-LS, QOL, QOR, AE,  MR, Crying/Laughing Episode rates |
|  |  |  |  | Enzyme Inhibitor | 33 | 22/12 | 55.3(9.5) | 1M |  |
| G. J. Groeneveld | 2003 | RCT | Netherlands | Dietary Supplements | 88 | 56/32 | 57.1(11.2) | 16M | MR, MVIC, ALSFRS, Arm megascore, VC, AE |
|  |  |  |  | Placebo | 87 | 62/25 | 58.4(10.9) | 16M |  |
| M. E. Cudkowicz | 2003 | RCT | US | Ion Channel Modulators | 197 | 125/72 | 57.8(12.4) | 12M | AE, MR, Arm megascore,  Grip megascore,FVC, ALSFRS |
|  |  |  |  | Placebo | 97 | 64/33 | 57.7(12.6) | 12M |  |
| G. Bensimon | 2002 | RCT | US | Neuroprotective Agent | 82 | 34/48 | 57.8(12.7) | 12M | MVIC, MR, AE |
|  |  |  |  | Placebo | 86 | 48/38 | 62.8(13.0) | 12M |  |
| R. G. Miller | 2001 | RCT | US | Ion Channel Modulators | 102 | 64/38 | 61.4(13.1) | 9M | MVIC, Arm megascore, FVC, MVC, MR, AE |
|  |  |  |  | Placebo | 100 | 59/41 | 62.0(12.0) | 9M |  |
| C. Desnuelle | 2001 | RCT | France | Antioxidants | 144 | 79/65 | 62.5(11.2) | 12M | Norris, MR, AHSS, AE, MR |
|  |  |  |  | Placebo | 144 | 79/65 | 65.7(9.4) | 12M |  |
| G. Ochs | 2000 | RCT | Germany,US | Cell Signaling Modulators | 20 | 14/6 | 57.1(5.6) | 12M | rHBDNF, MR, AE |
|  |  |  |  | Placebo | 5 | 4/1 | 59.5(10.5) | 12M |  |
| E. Beghi | 2000 | RCT | Italy | Immunomodulators | 31 | 23/8 | 57.5(6.5) | 6M | AE, Norris, MRC, FVC, MR |
|  |  |  |  | Placebo | 30 | 19/11 | 57.8(7.6) | 6M |  |

* SD was not provided in the original publication.
