# Supplementary material for: Efficacy and safety of pharmacological and biological therapies for amyotrophic lateral sclerosis: a network meta-analysis
Source: Front Neurol. 2026 Apr 24;17:1754716. doi: 10.3389/fneur.2026.1754716 (PMC13154608; doi:10.3389/fneur.2026.1754716)
Supplement: Supplementary file 2 [file Table_2.docx]

**Supplementary Table 2.** League table of mean differences (MD, 95% CrI) for ALSFRS-R between interventions.

| Placebo |  |  |  |  |  |  |  |  |  |  |  |  |  |  |  |  |  |  |  |  |  |  |
| --- | --- | --- | --- | --- | --- | --- | --- | --- | --- | --- | --- | --- | --- | --- | --- | --- | --- | --- | --- | --- | --- | --- |
| -0.21 (-3.03, 2.62) | Cytokine |  |  |  |  |  |  |  |  |  |  |  |  |  |  |  |  |  |  |  |  |  |
| -0.21 (-1.27, 0.85) | 0 (-3.02, 3.01) | Immunosuppressant |  |  |  |  |  |  |  |  |  |  |  |  |  |  |  |  |  |  |  |  |
| -0.85 (-2.59, 0.89) | -0.65 (-3.96, 2.67) | -0.65 (-2.67, 1.4) | Complement Inhibitor |  |  |  |  |  |  |  |  |  |  |  |  |  |  |  |  |  |  |  |
| 0.16 (-0.64, 0.96) | 0.37 (-2.58, 3.31) | 0.37 (-0.96, 1.7) | 1.01 (-0.91, 2.92) | Ion Channel Modulators |  |  |  |  |  |  |  |  |  |  |  |  |  |  |  |  |  |  |
| **1.01 (0.38, 1.64**) | 1.21 (-1.68, 4.11) | 1.22 (-0.01, 2.44) | **1.86 (0, 3.72)** | 0.85 (-0.18, 1.87) | Receptor Agonist |  |  |  |  |  |  |  |  |  |  |  |  |  |  |  |  |  |
| 0.55 (-1.48, 2.55) | 0.75 (-2.72, 4.21) | 0.76 (-1.52, 3.03) | 1.4 (-1.26, 4.06) | 0.39 (-1.79, 2.54) | -0.46 (-2.57, 1.64) | Receptor Antagonist |  |  |  |  |  |  |  |  |  |  |  |  |  |  |  |  |
| **1.15 (0.56, 1.74)** | 1.35 (-1.54, 4.24) | **1.36 (0.15, 2.57)** | **2 (0.17, 3.84)** | 0.99 (-0.01, 1.99) | 0.14 (-0.72, 1.01) | 0.6 (-1.49, 2.71) | Enzyme Inhibitor |  |  |  |  |  |  |  |  |  |  |  |  |  |  |  |
| **1.66 (0.32, 3)** | 1.87 (-1.26, 5) | **1.87 (0.17, 3.57)** | **2.52 (0.33, 4.7)** | 1.5 (-0.06, 3.06) | 0.66 (-0.82, 2.13) | 1.12 (-1.3, 3.54) | 0.51 (-0.94, 1.97) | Antioxidants |  |  |  |  |  |  |  |  |  |  |  |  |  |  |
| 0.15 (-0.35, 0.65) | 0.36 (-2.52, 3.22) | 0.36 (-0.82, 1.53) | 1 (-0.81, 2.81) | -0.01 (-0.96, 0.93) | **-0.86 (-1.66, -0.05)** | -0.4 (-2.46, 1.69) | **-1 (-1.78, -0.23)** | **-1.52 (-2.94, -0.08)** | Cell Signaling Modulators |  |  |  |  |  |  |  |  |  |  |  |  |  |
| 2.67 (-0.29, 5.62) | 2.88 (-1.22, 7) | 2.88 (-0.27, 6.03) | **3.53 (0.08, 6.95)** | 2.52 (-0.56, 5.58) | 1.67 (-1.37, 4.68) | 2.13 (-1.44, 5.69) | 1.53 (-1.5, 4.53) | 1.01 (-2.25, 4.25) | 2.53 (-0.48, 5.51) | ASO |  |  |  |  |  |  |  |  |  |  |  |  |
| 0.28 (-0.74, 1.31) | 0.49 (-2.51, 3.49) | 0.49 (-0.98, 1.96) | 1.13 (-0.89, 3.15) | 0.12 (-1.13, 1.38) | -0.73 (-1.92, 0.48) | -0.27 (-2.51, 2) | -0.87 (-2.04, 0.32) | -1.38 (-3.06, 0.3) | 0.13 (-1, 1.27) | -2.4 (-5.51, 0.74) | Neuroprotective Agent |  |  |  |  |  |  |  |  |  |  |  |
| 0.36 (-1.49, 2.22) | 0.57 (-2.81, 3.94) | 0.57 (-1.57, 2.69) | 1.22 (-1.33, 3.75) | 0.2 (-1.82, 2.22) | -0.64 (-2.6, 1.3) | -0.18 (-2.91, 2.57) | -0.79 (-2.73, 1.16) | -1.3 (-3.58, 0.97) | 0.21 (-1.71, 2.13) | -2.31 (-5.8, 1.2) | 0.08 (-2.04, 2.2) | Cell Therapy |  |  |  |  |  |  |  |  |  |  |
| **2.35 (1.14, 3.55)** | 2.55 (-0.51, 5.63) | **2.56 (0.95, 4.14)** | **3.2 (1.09, 5.31)** | **2.19 (0.74, 3.64)** | 1.34 (-0.02, 2.7) | 1.8 (-0.42, 4.06) | 1.2 (-0.14, 2.54) | 0.68 (-1.12, 2.49) | **2.2 (0.89, 3.5)** | -0.33 (-3.52, 2.87) | **2.06 (0.49, 3.65)** | 1.98 (-0.22, 4.2) | Nutritional Supplement |  |  |  |  |  |  |  |  |  |
| 1.95 (-0.69, 4.61) | 2.16 (-1.71, 6.07) | 2.16 (-0.69, 5.04) | 2.81 (-0.36, 5.98) | 1.8 (-0.96, 4.57) | 0.95 (-1.77, 3.69) | 1.41 (-1.91, 4.74) | 0.8 (-1.9, 3.54) | 0.29 (-2.67, 3.27) | 1.81 (-0.88, 4.51) | -0.72 (-4.69, 3.26) | 1.67 (-1.16, 4.52) | 1.6 (-1.64, 4.84) | -0.39 (-3.31, 2.54) | Alkaloid |  |  |  |  |  |  |  |  |
| 0.31 (-1.9, 2.52) | 0.52 (-3.09, 4.11) | 0.52 (-1.92, 2.97) | 1.16 (-1.65, 3.97) | 0.15 (-2.2, 2.51) | -0.7 (-3, 1.62) | -0.24 (-3.22, 2.77) | -0.84 (-3.14, 1.46) | -1.36 (-3.94, 1.25) | 0.16 (-2.11, 2.43) | -2.37 (-6.07, 1.35) | 0.03 (-2.42, 2.46) | -0.06 (-2.95, 2.85) | -2.04 (-4.55, 0.48) | -1.65 (-5.12, 1.8) | Microbial Therapeutics |  |  |  |  |  |  |  |
| -0.63 (-1.81, 0.55) | -0.42 (-3.48, 2.63) | -0.43 (-2, 1.17) | 0.22 (-1.87, 2.33) | -0.79 (-2.22, 0.64) | -1.64 (-2.97, -0.29) | -1.18 (-3.5, 1.16) | **-1.78 (-3.1, -0.46)** | **-2.29 (-4.08, -0.51)** | -0.78 (-2.07, 0.5) | **-3.31 (-6.48, -0.12)** | -0.91 (-2.48, 0.64) | -0.99 (-3.19, 1.21) | **-2.98 (-4.66, -1.29)** | -2.59 (-5.51, 0.32) | -0.94 (-3.45, 1.57) | Mood Stabilizer |  |  |  |  |  |  |
| 1.9 (-1.18, 4.98) | 2.1 (-2.09, 6.29) | 2.1 (-1.14, 5.36) | 2.76 (-0.78, 6.29) | 1.74 (-1.43, 4.92) | 0.89 (-2.24, 4.03) | 1.36 (-2.33, 5.02) | 0.75 (-2.37, 3.88) | 0.24 (-3.11, 3.58) | 1.75 (-1.36, 4.87) | -0.77 (-5.04, 3.5) | 1.62 (-1.63, 4.86) | 1.54 (-2.05, 5.13) | -0.45 (-3.75, 2.87) | -0.05 (-4.13, 4) | 1.59 (-2.18, 5.37) | 2.53 (-0.77, 5.81) | Chemically Modified Lipid Therapy |  |  |  |  |  |
| 0.79 (-1.1, 2.69) | 1 (-2.41, 4.42) | 1 (-1.17, 3.18) | 1.64 (-0.93, 4.23) | 0.63 (-1.43, 2.7) | -0.21 (-2.2, 1.79) | 0.24 (-2.51, 3.03) | -0.35 (-2.34, 1.63) | -0.87 (-3.19, 1.44) | 0.64 (-1.32, 2.61) | -1.88 (-5.4, 1.63) | 0.51 (-1.65, 2.67) | 0.43 (-2.23, 3.08) | -1.56 (-3.79, 0.69) | -1.16 (-4.43, 2.1) | 0.48 (-2.44, 3.41) | 1.42 (-0.81, 3.65) | -1.11 (-4.71, 2.51) | Nanomedicine |  |  |  |  |
| -0.6 (-2.11, 0.91) | -0.39 (-3.6, 2.82) | -0.39 (-2.22, 1.45) | 0.25 (-2.04, 2.56) | -0.75 (-2.46, 0.95) | -1.6 (-3.24, 0.03) | -1.15 (-3.65, 1.39) | **-1.74 (-3.37, -0.12)** | **-2.26 (-4.27, -0.24)** | -0.75 (-2.34, 0.84) | -3.27 (-6.6, 0.06) | -0.88 (-2.7, 0.94) | -0.96 (-3.36, 1.43) | -2.94 (-4.88, -1.02) | -2.55 (-5.6, 0.51) | -0.9 (-3.58, 1.77) | 0.04 (-1.88, 1.95) | -2.5 (-5.92, 0.93) | -1.39 (-3.81, 1.04) | Immunomodulators |  |  |  |
| **2.5 (1.15, 3.89)** | 2.71 (-0.42, 5.83) | **2.71 (0.99, 4.45)** | **3.35 (1.15, 5.57)** | **2.34 (0.77, 3.94)** | **1.49 (0, 3.02)** | 1.95 (-0.48, 4.4) | 1.35 (-0.13, 2.86) | 0.84 (-1.06, 2.76) | **2.35 (0.92, 3.82)** | -0.18 (-3.43, 3.11) | **2.22 (0.53, 3.93)** | 2.13 (-0.15, 4.45) | 0.15 (-1.68, 1.99) | 0.54 (-2.44, 3.54) | 2.19 (-0.42, 4.8) | **3.13 (1.33, 4.95)** | 0.6 (-2.78, 3.98) | 1.71 (-0.63, 4.05) | **3.09 (1.07, 5.14)** | Chinese Herbal Medicine |  |  |
| **3.32 (1.08, 5.56)** | 3.53 (-0.09, 7.12) | **3.53 (1.06, 6)** | **4.17 (1.34, 7.01)** | **3.16 (0.79, 5.54)** | **2.31 (0, 4.63)** | 2.77 (-0.25, 5.8) | 2.17 (-0.15, 4.48) | 1.66 (-0.96, 4.27) | **3.17 (0.88, 5.47)** | 0.65 (-3.04, 4.36) | **3.04 (0.59, 5.5)** | **2.96 (0.04, 5.85)** | 0.98 (-1.57, 3.52) | 1.36 (-2.11, 4.85) | 3.01 (-0.13, 6.15) | **3.95 (1.43, 6.48)** | 1.42 (-2.37, 5.24) | 2.53 (-0.42, 5.47) | **3.92 (1.23, 6.64)** | 0.82 (-1.81, 3.45) | Enzyme Inhibitor+Cell Signaling Modulators |  |
| **3.65 (1.27, 6.05)** | **3.86 (0.17, 7.55)** | **3.86 (1.25, 6.49)** | **4.51 (1.55, 7.46)** | **3.5 (1, 5.99)** | **2.65 (0.19, 5.13)** | **3.11 (0, 6.24)** | **2.5 (0.05, 4.97)** | 2 (-0.75, 4.74) | **3.51 (1.07, 5.96)** | 0.98 (-2.83, 4.8) | **3.37 (1.22, 5.54)** | **3.3 (0.27, 6.32)** | 1.31 (-1.37, 3.99) | 1.7 (-1.86, 5.27) | **3.35 (0.1, 6.61)** | **4.29 (1.63, 6.95)** | 1.75 (-2.12, 5.66) | 2.86 (-0.18, 5.91) | **4.25 (1.42, 7.08)** | 1.16 (-1.61, 3.89) | 0.33 (-2.94, 3.62) | Cell Therapy+Neuroprotective Agent |

*Note:* Each cell shows the MD (95% CrI) for the intervention in the row compared with that in the column. A higher MD indicates a better ALS Functional Rating Scale-Revised (ALSFRS-R) score. Bolded values denote statistically significant improvements.
