# Supplementary material for: Efficacy and safety of pharmacological and biological therapies for amyotrophic lateral sclerosis: a network meta-analysis
Source: Front Neurol. 2026 Apr 24;17:1754716. doi: 10.3389/fneur.2026.1754716 (PMC13154608; doi:10.3389/fneur.2026.1754716)
Supplement: Supplementary file 3 [file Table_3.docx]

**Supplementary Table 3.** League table of mean differences (MDs, 95% CrI) in FVC between interventions.

| Placebo |  |  |  |  |  |  |  |  |  |  |  |  |
| --- | --- | --- | --- | --- | --- | --- | --- | --- | --- | --- | --- | --- |
| **3.56 (2.34, 4.77)** | Immunosuppressant |  |  |  |  |  |  |  |  |  |  |  |
| **-10.27 (-13, -7.54)** | **-13.82 (-16.83, -10.83)** | Ion Channel Modulators |  |  |  |  |  |  |  |  |  |  |
| 18.52 (-2.48, 39.39) | 14.97 (-6.07, 35.87) | **28.79 (7.62, 49.82)** | Receptor Agonist |  |  |  |  |  |  |  |  |  |
| **-12.03 (-19.75, -4.33)** | **-15.58 (-23.37, -7.78)** | -1.76 (-9.95, 6.43) | **-30.53 (-52.78, -8.21)** | Receptor Antagonist |  |  |  |  |  |  |  |  |
| **2.82 (0.83, 4.82)** | -0.73 (-3.07, 1.62) | **13.1 (9.71, 16.49)** | -15.69 (-36.65, 5.4) | **14.86 (6.92, 22.85)** | Enzyme Inhibitor |  |  |  |  |  |  |  |
| **4.73 (2.02, 7.44)** | 1.18 (-1.81, 4.15) | **15.01 (11.16, 18.85)** | -13.77 (-34.89, 7.39) | **16.76 (8.61, 24.94)** | 1.9 (-1.45, 5.26) | Antioxidants |  |  |  |  |  |  |
| **9.59 (2.26, 16.85)** | 6.03 (-1.37, 13.41) | **19.86 (12.07, 27.61)** | -8.93 (-31.01, 13.22) | **21.63 (10.97, 32.22)** | 6.76 (-0.83, 14.26) | 4.86 (-2.95, 12.61) | Neuroprotective Agent |  |  |  |  |  |
| 2 (-3.01, 7.02) | -1.55 (-6.69, 3.62) | **12.28 (6.58, 17.97)** | -16.51 (-37.97, 4.98) | **14.05 (4.8, 23.21)** | -0.82 (-6.21, 4.56) | -2.73 (-8.42, 2.98) | -7.58 (-16.43, 1.25) | Nutritional Supplement |  |  |  |  |
| 3.7 (-6.8, 14.25) | 0.14 (-10.43, 10.76) | **13.97 (3.08, 24.85)** | -14.77 (-38.39, 8.65) | **15.74 (2.69, 28.74)** | 0.87 (-9.85, 11.59) | -1.03 (-11.89, 9.84) | -5.88 (-18.65, 6.96) | 1.71 (-10.03, 13.39) | Alkaloid |  |  |  |
| 1.26 (-2.05, 4.54) | -2.3 (-5.83, 1.2) | **11.53 (7.23, 15.79)** | -17.25 (-38.39, 3.96) | **13.29 (4.89, 21.65)** | -1.57 (-5.44, 2.27) | -3.47 (-7.77, 0.8) | **-8.34 (-16.33, -0.29)** | -0.76 (-6.74, 5.27) | -2.45 (-13.5, 8.59) | Microbial Therapeutics |  |  |
| **9.7 (0.81, 18.53)** | 6.14 (-2.83, 15.05) | **19.97 (10.66, 29.21)** | -8.79 (-31.53, 13.87) | **21.74 (9.97, 33.44)** | 6.88 (-2.27, 15.96) | 4.97 (-4.33, 14.23) | 0.1 (-11.37, 11.58) | 7.7 (-2.51, 17.86) | 5.98 (-7.78, 19.79) | 8.44 (-1.05, 17.9) | Nanomedicine |  |
| **9.07 (0.32, 17.77)** | 5.52 (-3.33, 14.31) | **19.33 (10.19, 28.46)** | -9.48 (-32.04, 13.27) | **21.1 (9.38, 32.79)** | 6.24 (-2.73, 15.15) | 4.34 (-4.79, 13.47) | -0.52 (-5.33, 4.31) | 7.07 (-3.03, 17.11) | 5.38 (-8.39, 19.03) | 7.82 (-1.55, 17.13) | -0.62 (-13.11, 11.81) | Cell Therapy+  Neuroprotective Agent |

*Note:* Each cell shows the mean difference (MD) with 95% credible intervals (CrIs) for the intervention in the row compared with that in the column. A higher MD indicates a higher forced vital capacity (FVC), while 0 indicates no difference between the two interventions. Bolded values represent statistically significant increases in FVC.
