# Supplementary material for: Efficacy and safety of pharmacological and biological therapies for amyotrophic lateral sclerosis: a network meta-analysis
Source: Front Neurol. 2026 Apr 24;17:1754716. doi: 10.3389/fneur.2026.1754716 (PMC13154608; doi:10.3389/fneur.2026.1754716)
Supplement: Supplementary file 4 [file Table_4.docx]

**Supplementary Table 4.** League table of relative risks (RRs, 95% CrI) for mortality between interventions.

| Placebo |  |  |  |  |  |  |  |  |  |  |  |  |  |  |  |  |  |  |  |  |  |  |  |  |  |
| --- | --- | --- | --- | --- | --- | --- | --- | --- | --- | --- | --- | --- | --- | --- | --- | --- | --- | --- | --- | --- | --- | --- | --- | --- | --- |
| 1 (0.81, 1.24) | Cytokine |  |  |  |  |  |  |  |  |  |  |  |  |  |  |  |  |  |  |  |  |  |  |  |  |
| 1.25 (0.49, 3.39) | 1.25 (0.48, 3.46) | Immunosuppressant |  |  |  |  |  |  |  |  |  |  |  |  |  |  |  |  |  |  |  |  |  |  |  |
| 0.97 (0.49, 1.94) | 0.97 (0.48, 2.01) | 0.77 (0.23, 2.47) | Complement Inhibitor |  |  |  |  |  |  |  |  |  |  |  |  |  |  |  |  |  |  |  |  |  |  |
| 0.91 (0.6, 1.4) | 0.91 (0.57, 1.47) | 0.73 (0.25, 2.03) | 0.95 (0.42, 2.1) | Ion Channel Modulators |  |  |  |  |  |  |  |  |  |  |  |  |  |  |  |  |  |  |  |  |  |
| 1.02 (0.76, 1.38) | 1.02 (0.71, 1.48) | 0.82 (0.29, 2.17) | 1.06 (0.5, 2.21) | 1.12 (0.67, 1.87) | Receptor Agonist |  |  |  |  |  |  |  |  |  |  |  |  |  |  |  |  |  |  |  |  |
| 1.04 (0.76, 1.42) | 1.04 (0.71, 1.52) | 0.83 (0.29, 2.2) | 1.08 (0.5, 2.27) | 1.14 (0.67, 1.93) | 1.02 (0.66, 1.57) | Receptor Antagonist |  |  |  |  |  |  |  |  |  |  |  |  |  |  |  |  |  |  |  |
| 1 (0.91, 1.1) | 1 (0.79, 1.26) | 0.8 (0.29, 2.03) | 1.03 (0.51, 2.05) | 1.1 (0.71, 1.68) | 0.98 (0.72, 1.33) | 0.96 (0.69, 1.33) | Enzyme Inhibitor |  |  |  |  |  |  |  |  |  |  |  |  |  |  |  |  |  |  |
| 0.86 (0.61, 1.19) | 0.86 (0.58, 1.27) | 0.69 (0.24, 1.84) | 0.89 (0.41, 1.88) | 0.94 (0.55, 1.6) | 0.84 (0.54, 1.31) | 0.82 (0.52, 1.3) | 0.86 (0.61, 1.21) | Antioxidants |  |  |  |  |  |  |  |  |  |  |  |  |  |  |  |  |  |
| 0.83 (0.59, 1.18) | 0.83 (0.56, 1.26) | 0.67 (0.23, 1.79) | 0.86 (0.4, 1.85) | 0.91 (0.53, 1.57) | 0.82 (0.52, 1.29) | 0.8 (0.5, 1.28) | 0.83 (0.58, 1.19) | 0.97 (0.61, 1.57) | Cell Signaling Modulators |  |  |  |  |  |  |  |  |  |  |  |  |  |  |  |  |
| 0.57 (0.17, 2.23) | 0.58 (0.16, 2.28) | 0.46 (0.09, 2.33) | 0.6 (0.15, 2.7) | 0.63 (0.17, 2.62) | 0.56 (0.16, 2.26) | 0.55 (0.15, 2.22) | 0.58 (0.17, 2.24) | 0.67 (0.19, 2.71) | 0.69 (0.19, 2.8) | ASO |  |  |  |  |  |  |  |  |  |  |  |  |  |  |  |
| 0.92 (0.81, 1.03) | 0.92 (0.72, 1.17) | 0.73 (0.27, 1.87) | 0.95 (0.47, 1.88) | 1 (0.65, 1.55) | 0.9 (0.65, 1.24) | 0.88 (0.63, 1.23) | 0.92 (0.79, 1.07) | 1.07 (0.75, 1.52) | 1.1 (0.76, 1.58) | 1.59 (0.41, 5.51) | Neuroprotective Agent |  |  |  |  |  |  |  |  |  |  |  |  |  |  |
| 1.43 (0.57, 3.84) | 1.43 (0.55, 3.92) | 1.15 (0.3, 4.43) | 1.49 (0.47, 4.85) | 1.57 (0.57, 4.55) | 1.41 (0.53, 3.92) | 1.38 (0.52, 3.87) | 1.43 (0.57, 3.87) | 1.67 (0.63, 4.72) | 1.72 (0.64, 4.9) | 2.5 (0.49, 12.18) | 1.56 (0.62, 4.22) | Cell Therapy |  |  |  |  |  |  |  |  |  |  |  |  |  |
| 0.89 (0.73, 1.08) | 0.89 (0.67, 1.19) | 0.71 (0.26, 1.84) | 0.92 (0.45, 1.86) | 0.97 (0.61, 1.55) | 0.87 (0.61, 1.24) | 0.85 (0.59, 1.23) | 0.89 (0.71, 1.1) | 1.04 (0.71, 1.53) | 1.07 (0.72, 1.58) | 1.54 (0.39, 5.4) | 0.97 (0.77, 1.22) | 0.62 (0.23, 1.59) | Nutritional Supplement |  |  |  |  |  |  |  |  |  |  |  |  |
| 0.5 (0.1, 2.64) | 0.5 (0.09, 2.7) | 0.4 (0.06, 2.68) | 0.52 (0.09, 3.14) | 0.55 (0.1, 3.03) | 0.49 (0.09, 2.66) | 0.48 (0.09, 2.61) | 0.5 (0.1, 2.65) | 0.59 (0.11, 3.18) | 0.6 (0.11, 3.28) | 0.87 (0.1, 6.89) | 0.55 (0.1, 2.89) | 0.35 (0.05, 2.31) | 0.57 (0.11, 3.01) | Alkaloid |  |  |  |  |  |  |  |  |  |  |  |
| 0.92 (0.03, 31.44) | 0.92 (0.03, 31.66) | 0.74 (0.02, 27.75) | 0.95 (0.02, 34.19) | 1.01 (0.03, 35.05) | 0.9 (0.02, 31.29) | 0.89 (0.02, 30.5) | 0.92 (0.03, 31.46) | 1.07 (0.03, 37.07) | 1.11 (0.03, 38.34) | 1.59 (0.03, 66.19) | 1.01 (0.03, 34.32) | 0.64 (0.02, 24.16) | 1.04 (0.03, 35.4) | 1.83 (0.04, 87.4) | Microbial Therapeutics |  |  |  |  |  |  |  |  |  |  |
| 1.16 (0.94, 1.43) | 1.16 (0.86, 1.56) | 0.93 (0.34, 2.39) | 1.2 (0.58, 2.43) | 1.27 (0.79, 2.03) | 1.14 (0.79, 1.63) | 1.11 (0.77, 1.62) | 1.16 (0.92, 1.46) | 1.35 (0.91, 2) | 1.4 (0.93, 2.08) | 2.02 (0.51, 7.04) | 1.27 (0.99, 1.61) | 0.81 (0.29, 2.08) | 1.3 (0.98, 1.74) | 2.3 (0.43, 12.32) | 1.26 (0.04, 46.18) | Mood Stabilizer |  |  |  |  |  |  |  |  |  |
| 1.02 (0.03, 35.78) | 1.02 (0.03, 36.18) | 0.81 (0.02, 31.76) | 1.05 (0.03, 39.07) | 1.11 (0.03, 40.12) | 1 (0.03, 35.5) | 0.98 (0.03, 34.73) | 1.02 (0.03, 35.76) | 1.19 (0.03, 42.8) | 1.22 (0.03, 43.35) | 1.75 (0.04, 75.18) | 1.11 (0.03, 38.77) | 0.71 (0.02, 27.7) | 1.15 (0.03, 40.57) | 2.03 (0.04, 98.62) | 1.11 (0.01, 162.33) | 0.88 (0.02, 30.83) | Chemically Modified Lipid Therapy |  |  |  |  |  |  |  |  |
| 0.6 (0.07, 3.77) | 0.6 (0.07, 3.83) | 0.48 (0.05, 3.77) | 0.62 (0.07, 4.39) | 0.66 (0.08, 4.36) | 0.59 (0.07, 3.8) | 0.58 (0.07, 3.74) | 0.6 (0.07, 3.79) | 0.7 (0.08, 4.53) | 0.72 (0.08, 4.69) | 1.03 (0.09, 9.51) | 0.66 (0.08, 4.15) | 0.41 (0.04, 3.29) | 0.68 (0.08, 4.31) | 1.18 (0.08, 14.25) | 0.64 (0.01, 35.32) | 0.52 (0.06, 3.3) | 0.58 (0.01, 33.29) | Nanomedicine |  |  |  |  |  |  |  |
| 1.19 (0.68, 2.1) | 1.19 (0.65, 2.19) | 0.95 (0.3, 2.82) | 1.23 (0.5, 2.98) | 1.3 (0.64, 2.64) | 1.17 (0.62, 2.21) | 1.14 (0.6, 2.2) | 1.19 (0.67, 2.12) | 1.39 (0.72, 2.69) | 1.43 (0.74, 2.77) | 2.07 (0.47, 8.08) | 1.3 (0.73, 2.33) | 0.83 (0.27, 2.47) | 1.34 (0.74, 2.45) | 2.36 (0.41, 13.7) | 1.29 (0.04, 48.99) | 1.03 (0.56, 1.89) | 1.17 (0.03, 45.2) | 1.99 (0.29, 17.51) | Immunomodulators |  |  |  |  |  |  |
| 0.64 (0.08, 3.82) | 0.64 (0.08, 3.87) | 0.5 (0.05, 3.81) | 0.65 (0.07, 4.47) | 0.7 (0.08, 4.37) | 0.62 (0.07, 3.85) | 0.61 (0.07, 3.79) | 0.64 (0.08, 3.82) | 0.74 (0.09, 4.57) | 0.76 (0.09, 4.76) | 1.08 (0.09, 9.76) | 0.7 (0.08, 4.18) | 0.44 (0.04, 3.34) | 0.72 (0.09, 4.35) | 1.25 (0.09, 14.8) | 0.67 (0.01, 36.59) | 0.55 (0.07, 3.32) | 0.61 (0.01, 34.73) | 1.05 (0.07, 16.96) | 0.53 (0.06, 3.51) | Chinese Herbal Medicine |  |  |  |  |  |
| 1 (0.03, 33.19) | 1 (0.03, 33.67) | 0.79 (0.02, 29.97) | 1.03 (0.03, 37.02) | 1.09 (0.03, 37.7) | 0.98 (0.03, 33.11) | 0.96 (0.03, 32.36) | 1 (0.03, 33.3) | 1.17 (0.03, 39.42) | 1.2 (0.03, 41.12) | 1.71 (0.04, 71.49) | 1.09 (0.03, 36.27) | 0.69 (0.02, 26.06) | 1.12 (0.03, 37.91) | 1.98 (0.04, 95.37) | 1.09 (0.01, 152.06) | 0.86 (0.02, 28.92) | 0.98 (0.01, 141.77) | 1.68 (0.03, 96.13) | 0.84 (0.02, 29.38) | 1.61 (0.03, 91.43) | Radiation Therapy |  |  |  |  |
| 0.52 (0.01, 18.26) | 0.52 (0.01, 18.32) | 0.41 (0.01, 16.19) | 0.54 (0.01, 19.97) | 0.57 (0.02, 20.35) | 0.51 (0.01, 18.12) | 0.5 (0.01, 17.55) | 0.52 (0.01, 18.29) | 0.61 (0.02, 21.4) | 0.63 (0.02, 22.2) | 0.89 (0.02, 38.42) | 0.57 (0.02, 19.88) | 0.36 (0.01, 14.13) | 0.59 (0.02, 20.62) | 1.03 (0.02, 51.87) | 0.56 (0, 80.21) | 0.45 (0.01, 15.89) | 0.51 (0, 78.27) | 0.89 (0.02, 52.66) | 0.44 (0.01, 15.81) | 0.84 (0.02, 49.97) | 0.52 (0, 74.3) | Receptor Modulator+Enzyme Inhibitor |  |  |  |
| 0.91 (0.03, 31.21) | 0.91 (0.03, 31.25) | 0.73 (0.02, 27.47) | 0.94 (0.03, 34.2) | 1 (0.03, 34.85) | 0.9 (0.02, 30.81) | 0.88 (0.02, 30.22) | 0.92 (0.03, 31.22) | 1.07 (0.03, 36.92) | 1.1 (0.03, 38.3) | 1.57 (0.04, 65.01) | 1 (0.03, 34.02) | 0.63 (0.02, 24.24) | 1.03 (0.03, 35.19) | 1.82 (0.04, 86.17) | 0.99 (0.01, 146.45) | 0.79 (0.02, 26.87) | 0.9 (0.01, 132.05) | 1.56 (0.03, 90.95) | 0.77 (0.02, 27.21) | 1.47 (0.03, 84.89) | 0.91 (0.01, 123.23) | 1.76 (0.01, 254.64) | Free Radical Scavenger+Neuroprotective Agent |  |  |
| 1.53 (0.32, 11.76) | 1.53 (0.32, 11.88) | 1.23 (0.19, 11.44) | 1.59 (0.29, 13.43) | 1.68 (0.33, 13.37) | 1.5 (0.31, 11.75) | 1.47 (0.3, 11.53) | 1.53 (0.32, 11.8) | 1.79 (0.36, 14.05) | 1.84 (0.37, 14.53) | 2.7 (0.34, 28.62) | 1.67 (0.35, 12.9) | 1.08 (0.17, 9.79) | 1.72 (0.36, 13.4) | 3.14 (0.31, 41.48) | 1.73 (0.04, 98.9) | 1.32 (0.27, 10.29) | 1.58 (0.03, 92.61) | 2.65 (0.23, 45.89) | 1.29 (0.24, 10.73) | 2.52 (0.22, 44.28) | 1.61 (0.03, 88.67) | 3.08 (0.06, 181.46) | 1.74 (0.04, 101.58) | Enzyme Inhibitor+Cell Signaling Modulators |  |
| 0.85 (0.02, 29.96) | 0.85 (0.02, 30.34) | 0.68 (0.02, 26.7) | 0.88 (0.02, 32.4) | 0.93 (0.02, 33.59) | 0.83 (0.02, 29.92) | 0.82 (0.02, 29.14) | 0.85 (0.02, 30.1) | 0.99 (0.03, 35.38) | 1.02 (0.03, 36.71) | 1.46 (0.03, 62.88) | 0.93 (0.02, 32.8) | 0.59 (0.01, 23.77) | 0.96 (0.03, 33.78) | 1.69 (0.03, 83.68) | 0.92 (0.01, 133.85) | 0.73 (0.02, 25.99) | 0.84 (0.01, 127.36) | 1.44 (0.03, 85.1) | 0.71 (0.02, 26.26) | 1.37 (0.02, 81.02) | 0.85 (0.01, 125.19) | 1.63 (0.01, 247.8) | 0.93 (0.01, 136.96) | 0.53 (0.01, 26.56) | Cell Therapy+Neuroprotective Agent |

*Note:* Each cell shows the relative risk (RR) with 95% credible intervals (CrIs) for the intervention in the row compared with that in the column. An RR > 1 indicates a higher mortality rate, while an RR=1 indicates no difference between the two interventions. Bolded values represent statistically significant reductions in mortality.
