# Supplementary material for: Efficacy and safety of pharmacological and biological therapies for amyotrophic lateral sclerosis: a network meta-analysis
Source: Front Neurol. 2026 Apr 24;17:1754716. doi: 10.3389/fneur.2026.1754716 (PMC13154608; doi:10.3389/fneur.2026.1754716)
Supplement: Supplementary file 5 [file Table_5.docx]

**Supplementary Table 5.** League table of relative risks (RRs, 95% CrI) for serious adverse events between interventions.

| Placebo |  |  |  |  |  |  |  |  |  |  |  |  |  |  |  |  |  |  |  |  |  |  |  |  |
| --- | --- | --- | --- | --- | --- | --- | --- | --- | --- | --- | --- | --- | --- | --- | --- | --- | --- | --- | --- | --- | --- | --- | --- | --- |
| 1.44 (0.74, 2.91) | Cytokine |  |  |  |  |  |  |  |  |  |  |  |  |  |  |  |  |  |  |  |  |  |  |  |
| 1.17 (0.68, 2.08) | 0.81 (0.34, 1.98) | Immunosuppressant |  |  |  |  |  |  |  |  |  |  |  |  |  |  |  |  |  |  |  |  |  |  |
| 1.31 (0.9, 1.93) | 0.91 (0.41, 1.98) | 1.11 (0.56, 2.18) | Complement Inhibitor |  |  |  |  |  |  |  |  |  |  |  |  |  |  |  |  |  |  |  |  |  |
| 1.17 (0.9, 1.55) | 0.82 (0.38, 1.68) | 1 (0.53, 1.83) | 0.9 (0.56, 1.42) | Ion Channel Modulators |  |  |  |  |  |  |  |  |  |  |  |  |  |  |  |  |  |  |  |  |
| 1.12 (0.81, 1.55) | 0.78 (0.36, 1.64) | 0.95 (0.49, 1.8) | 0.86 (0.51, 1.41) | 0.95 (0.62, 1.45) | Receptor Agonist |  |  |  |  |  |  |  |  |  |  |  |  |  |  |  |  |  |  |  |
| 1.4 (0.97, 2.03) | 0.97 (0.44, 2.09) | 1.19 (0.6, 2.3) | 1.07 (0.62, 1.81) | 1.19 (0.75, 1.88) | 1.25 (0.77, 2.04) | Receptor Antagonist |  |  |  |  |  |  |  |  |  |  |  |  |  |  |  |  |  |  |
| **1.12 (1.01, 1.25)** | 0.78 (0.38, 1.53) | 0.96 (0.53, 1.66) | 0.86 (0.57, 1.26) | 0.96 (0.71, 1.27) | 1 (0.71, 1.41) | 0.8 (0.55, 1.18) | Enzyme Inhibitor |  |  |  |  |  |  |  |  |  |  |  |  |  |  |  |  |  |
| 1.09 (0.9, 1.34) | 0.76 (0.37, 1.53) | 0.93 (0.51, 1.67) | 0.84 (0.54, 1.28) | 0.93 (0.66, 1.3) | 0.98 (0.67, 1.43) | 0.78 (0.51, 1.19) | 0.97 (0.78, 1.22) | Antioxidants |  |  |  |  |  |  |  |  |  |  |  |  |  |  |  |  |
| 0.88 (0.73, 1.06) | 0.61 (0.29, 1.22) | 0.75 (0.41, 1.33) | 0.67 (0.44, 1.02) | 0.75 (0.54, 1.03) | 0.78 (0.54, 1.14) | 0.63 (0.41, 0.95) | 0.78 (0.63, 0.97) | 0.8 (0.61, 1.05) | Cell Signaling Modulators |  |  |  |  |  |  |  |  |  |  |  |  |  |  |  |
| 0.78 (0.45, 1.45) | 0.55 (0.22, 1.35) | 0.67 (0.3, 1.52) | 0.6 (0.3, 1.23) | 0.67 (0.36, 1.3) | 0.7 (0.37, 1.4) | 0.56 (0.29, 1.14) | 0.7 (0.39, 1.3) | 0.72 (0.39, 1.36) | 0.89 (0.49, 1.7) | ASO |  |  |  |  |  |  |  |  |  |  |  |  |  |  |
| 0.95 (0.87, 1.03) | 0.66 (0.32, 1.29) | 0.81 (0.45, 1.4) | 0.72 (0.49, 1.06) | 0.81 (0.61, 1.06) | 0.85 (0.6, 1.18) | 0.68 (0.46, 0.99) | 0.84 (0.74, 0.96) | 0.86 (0.69, 1.07) | 1.08 (0.88, 1.32) | 1.21 (0.65, 2.13) | Neuroprotective Agent |  |  |  |  |  |  |  |  |  |  |  |  |  |
| 1.37 (0.83, 2.35) | 0.95 (0.4, 2.24) | 1.17 (0.54, 2.5) | 1.05 (0.55, 2.02) | 1.17 (0.65, 2.13) | 1.23 (0.67, 2.29) | 0.98 (0.52, 1.88) | 1.22 (0.73, 2.11) | 1.25 (0.73, 2.22) | 1.56 (0.91, 2.76) | 1.74 (0.79, 3.81) | 1.45 (0.87, 2.5) | Cell Therapy |  |  |  |  |  |  |  |  |  |  |  |  |
| 1.02 (0.85, 1.21) | 0.71 (0.34, 1.41) | 0.87 (0.47, 1.53) | 0.78 (0.51, 1.17) | 0.87 (0.62, 1.19) | 0.91 (0.63, 1.31) | 0.73 (0.48, 1.09) | 0.9 (0.74, 1.11) | 0.93 (0.71, 1.21) | 1.16 (0.9, 1.5) | 1.3 (0.68, 2.33) | 1.07 (0.88, 1.31) | 0.74 (0.42, 1.27) | Nutritional Supplement |  |  |  |  |  |  |  |  |  |  |  |
| 1.04 (0.47, 2.66) | 0.72 (0.25, 2.28) | 0.89 (0.33, 2.6) | 0.8 (0.33, 2.17) | 0.88 (0.38, 2.35) | 0.93 (0.39, 2.48) | 0.74 (0.31, 2.04) | 0.92 (0.42, 2.38) | 0.95 (0.42, 2.47) | 1.18 (0.53, 3.08) | 1.33 (0.49, 3.97) | 1.1 (0.5, 2.83) | 0.76 (0.29, 2.2) | 1.02 (0.46, 2.64) | Alkaloid |  |  |  |  |  |  |  |  |  |  |
| 0.93 (0.03, 30.86) | 0.64 (0.02, 22.76) | 0.79 (0.02, 27.56) | 0.71 (0.02, 23.94) | 0.79 (0.02, 26.38) | 0.83 (0.02, 27.92) | 0.66 (0.02, 22.47) | 0.83 (0.02, 27.42) | 0.85 (0.02, 28.2) | 1.06 (0.03, 35.23) | 1.18 (0.03, 40.53) | 0.98 (0.03, 32.6) | 0.67 (0.02, 23.17) | 0.91 (0.03, 30.36) | 0.88 (0.02, 31.77) | Microbial Therapeutics |  |  |  |  |  |  |  |  |  |
| 1.07 (0.9, 1.29) | 0.74 (0.36, 1.49) | 0.91 (0.5, 1.62) | 0.82 (0.53, 1.24) | 0.91 (0.66, 1.26) | 0.96 (0.66, 1.39) | 0.77 (0.51, 1.16) | 0.95 (0.77, 1.17) | 0.98 (0.75, 1.28) | 1.22 (0.94, 1.58) | 1.37 (0.72, 2.46) | 1.13 (0.93, 1.39) | 0.78 (0.44, 1.34) | 1.05 (0.82, 1.36) | 1.03 (0.4, 2.31) | 1.16 (0.03, 41.92) | Mood Stabilizer |  |  |  |  |  |  |  |  |
| 2.49 (0.21, 70.81) | 1.73 (0.13, 51.53) | 2.12 (0.17, 62.33) | 1.9 (0.16, 55.16) | 2.12 (0.17, 60.35) | 2.23 (0.18, 64.28) | 1.78 (0.14, 51.66) | 2.22 (0.19, 63.21) | 2.27 (0.19, 65.4) | 2.84 (0.24, 81.41) | 3.17 (0.25, 93.3) | 2.63 (0.22, 75.03) | 1.82 (0.14, 52.98) | 2.45 (0.2, 69.95) | 2.39 (0.17, 73.3) | 2.88 (0.04, 342.68) | 2.32 (0.19, 66.52) |  |  |  |  |  |  |  |  |
| 0.97 (0.42, 2.23) | 0.67 (0.23, 1.96) | 0.83 (0.3, 2.23) | 0.74 (0.3, 1.85) | 0.83 (0.34, 1.99) | 0.87 (0.35, 2.12) | 0.69 (0.28, 1.73) | 0.86 (0.37, 2) | 0.89 (0.38, 2.09) | 1.11 (0.47, 2.6) | 1.23 (0.44, 3.39) | 1.03 (0.44, 2.37) | 0.71 (0.26, 1.87) | 0.96 (0.41, 2.24) | 0.93 (0.27, 2.94) | 1.05 (0.03, 41.65) | 0.91 (0.39, 2.12) | Chemically Modified Lipid Therapy |  |  |  |  |  |  |  |
| 1.19 (0.88, 1.62) | 0.83 (0.38, 1.73) | 1.01 (0.53, 1.89) | 0.91 (0.56, 1.48) | 1.01 (0.68, 1.53) | 1.07 (0.68, 1.66) | 0.85 (0.53, 1.37) | 1.06 (0.77, 1.47) | 1.09 (0.76, 1.57) | 1.36 (0.95, 1.94) | 1.52 (0.76, 2.89) | 1.26 (0.92, 1.73) | 0.87 (0.47, 1.57) | 1.17 (0.82, 1.67) | 1.15 (0.43, 2.67) | 1.28 (0.04, 47.19) | 1.11 (0.78, 1.59) | 0.39 (0.01, 5.27) | Nanomedicine |  |  |  |  |  |  |
| 1 (0.11, 9.27) | 0.7 (0.07, 7.01) | 0.85 (0.09, 8.44) | 0.77 (0.08, 7.28) | 0.85 (0.09, 7.97) | 0.9 (0.1, 8.43) | 0.72 (0.08, 6.8) | 0.89 (0.1, 8.26) | 0.92 (0.1, 8.53) | 1.15 (0.13, 10.59) | 1.27 (0.13, 12.65) | 1.06 (0.12, 9.78) | 0.73 (0.08, 7.12) | 0.99 (0.11, 9.15) | 0.95 (0.09, 10.11) | 1.09 (0.02, 70.66) | 0.94 (0.1, 8.71) | 0.48 (0.02, 5.83) | 1.23 (0.51, 2.97) | Immunomodulators |  |  |  |  |  |
| 1.11 (0.03, 38.74) | 0.77 (0.02, 28.47) | 0.94 (0.03, 33.98) | 0.85 (0.02, 30.21) | 0.95 (0.03, 33.52) | 0.99 (0.03, 35.34) | 0.79 (0.02, 28.31) | 0.99 (0.03, 34.4) | 1.01 (0.03, 35.39) | 1.27 (0.04, 44.06) | 1.41 (0.04, 50.34) | 1.18 (0.03, 40.8) | 0.81 (0.02, 29.21) | 1.09 (0.03, 38.05) | 1.05 (0.03, 39.8) | 1.2 (0.01, 173.83) | 1.04 (0.03, 35.75) | 0.39 (0.01, 11.06) | 1.04 (0.1, 10.95) | 0.84 (0.09, 7.92) | Chinese Herbal Medicine |  |  |  |  |
| **5.91 (1.69, 39.83)** | 4.13 (0.96, 30.53) | **5.04 (1.28, 36.55)** | **4.52 (1.21, 31.37)** | **5.04 (1.39, 34.35)** | **5.3 (1.45, 36.72)** | **4.25 (1.14, 29.54)** | **5.25 (1.51, 35.41)** | **5.4 (1.51, 36.73)** | **6.75 (1.9, 45.82)** | **7.57 (1.85, 54.5)** | **6.25 (1.78, 42.31)** | **4.35 (1.1, 31.12)** | **5.83 (1.64, 39.48)** | **5.69 (1.19, 44.03)** | 6.84 (0.16, 346.25) | **5.51 (1.56, 37.32)** | 0.42 (0, 31.79) | 1.14 (0.03, 43.67) | 0.93 (0.03, 32.83) | 1.1 (0.02, 68.69) | Radiation Therapy |  |  |  |
| 0.95 (0.03, 34.1) | 0.66 (0.02, 25.38) | 0.8 (0.02, 30.28) | 0.73 (0.02, 26.6) | 0.81 (0.02, 29.53) | 0.85 (0.02, 30.94) | 0.68 (0.02, 24.91) | 0.84 (0.02, 30.4) | 0.86 (0.02, 31.29) | 1.08 (0.03, 39.22) | 1.21 (0.03, 45.1) | 1 (0.03, 36) | 0.69 (0.02, 25.96) | 0.93 (0.03, 33.73) | 0.9 (0.02, 35.43) | 1.03 (0.01, 154.88) | 0.89 (0.02, 32.17) | 2.45 (0.07, 53.11) | **6.19 (1.33, 47.71)** | **4.97 (1.36, 34.03)** | 6.2 (0.46, 103.89) | 5.68 (0.12, 289.89) | Receptor Modulator+Enzyme Inhibitor |  |  |
| 0.68 (0.3, 1.6) | 0.47 (0.16, 1.4) | 0.58 (0.21, 1.59) | 0.52 (0.21, 1.32) | 0.58 (0.24, 1.42) | 0.61 (0.25, 1.52) | 0.49 (0.2, 1.24) | 0.6 (0.27, 1.44) | 0.62 (0.27, 1.5) | 0.78 (0.34, 1.86) | 0.86 (0.31, 2.41) | 0.72 (0.32, 1.7) | 0.49 (0.19, 1.34) | 0.67 (0.29, 1.6) | 0.65 (0.19, 2.11) | 0.73 (0.02, 29.33) | 0.63 (0.27, 1.52) | 0.36 (0, 29.1) | 0.98 (0.03, 38.41) | 0.8 (0.02, 28.91) | 0.95 (0.01, 61.52) | 0.86 (0.01, 124.54) | 0.15 (0, 6.91) | Free Radical Scavenger+Neuroprotective Agent |  |
| 0.42 (0.09, 1.51) | 0.29 (0.05, 1.24) | 0.35 (0.07, 1.44) | 0.32 (0.07, 1.23) | 0.36 (0.07, 1.32) | 0.38 (0.08, 1.41) | 0.3 (0.06, 1.14) | 0.37 (0.08, 1.35) | 0.38 (0.08, 1.4) | 0.48 (0.1, 1.75) | 0.53 (0.1, 2.17) | 0.44 (0.09, 1.6) | 0.3 (0.06, 1.21) | 0.41 (0.09, 1.51) | 0.4 (0.07, 1.83) | 0.44 (0.01, 19.62) | 0.39 (0.08, 1.43) | 0.27 (0.01, 3.78) | 0.7 (0.22, 2.29) | 0.57 (0.24, 1.41) | 0.68 (0.06, 7.11) | 0.61 (0.02, 23.24) | 0.11 (0.01, 0.54) | 0.72 (0.02, 27.63) | Enzyme Inhibitor+Cell Signaling Modulators |

*Note:* Each cell shows the relative risk (RR) with 95% credible intervals (CrIs) for the intervention in the row compared with that in the column. An RR > 1 indicates a higher incidence of serious adverse events, while an RR=1 indicates no difference between the two interventions. Bolded values represent statistically significant increases in serious adverse events.
