# Supplementary material for: Efficacy and safety of pharmacological and biological therapies for amyotrophic lateral sclerosis: a network meta-analysis
Source: Front Neurol. 2026 Apr 24;17:1754716. doi: 10.3389/fneur.2026.1754716 (PMC13154608; doi:10.3389/fneur.2026.1754716)
Supplement: Supplementary file 6 [file Data_Sheet_1.pdf]

Table S1. The search strategy (Pubmed)

Search number	Query	Results
1	Amyotrophic Lateral Sclerosis[MeSH Terms]	25,331
2	"ALS amyotrophic lateral sclerosis"[Title/Abstract] OR "als dementia"[Title/Abstract] OR "ALS Amyotrophic Lateral Sclerosis"[Title/Abstract] OR "Amyotrophic Lateral Sclerosis"[Title/Abstract] OR "Amyotrophic Lateral Sclerosis Parkinsonism Dementia Complex 1"[Title/Abstract] OR "Amyotrophic Lateral Sclerosis With Dementia"[Title/Abstract] OR "Charcot Disease"[Title/Abstract] OR "Dementia With Amyotrophic Lateral Sclerosis"[Title/Abstract] OR "Gehrig Disease"[Title/Abstract] OR "Gehrigs Disease"[Title/Abstract] OR "Guam Disease"[Title/Abstract] OR "Guam Form of Amyotrophic Lateral Sclerosis"[Title/Abstract] OR "Lou Gehrig Disease"[Title/Abstract] OR "Lou Gehrigs Disease"[Title/Abstract]	33,266
3	"randomised controlled study"[Title/Abstract] OR "randomised controlled trial"[Title/Abstract] OR "randomized controlled study"[Title/Abstract] OR "randomized controlled trial"[Title/Abstract] OR "random*"[Title/Abstract] OR "trial, randomized controlled"[Title/Abstract] OR "Clinical Trials, Randomized"[Title/Abstract] OR "Trials, Randomized Clinical"[Title/Abstract]	1,605,101
4	(#1 OR #2) AND #3	1,235

Table S2. The search strategy (Cochrane)

Search number	Query	Results
1	MeSH descriptor: [Amyotrophic Lateral Sclerosis] explode all trees	889
2	(‘ALS amyotrophic lateral sclerosis’ OR ‘als dementia’ OR ‘ALS Amyotrophic Lateral Sclerosis’ OR ‘Amyotrophic Lateral Sclerosis’ OR ‘Amyotrophic Lateral Sclerosis Parkinsonism Dementia Complex 1’ OR ‘Amyotrophic Lateral Sclerosis With Dementia’ OR ‘Charcot Disease’ OR ‘Dementia With Amyotrophic Lateral Sclerosis’ OR ‘Gehrig Disease’ OR ‘Gehrigs Disease’ OR ‘Guam Disease’ OR ‘Guam Form of Amyotrophic Lateral Sclerosis’ OR ‘Lou Gehrig Disease’ OR ‘Lou Gehrigs Disease’):ti,ab,kw	2028
3	(‘randomised controlled study’ OR ‘randomised controlled trial’ OR ‘randomized controlled study’ OR ‘randomized controlled trial’ OR ‘random*’ OR ‘trial, randomized controlled’ OR ‘Clinical Trials, Randomized’ OR ‘Trials, Randomized Clinical’):ti,ab,kw	1370177
4	(#1 OR #2) AND #3	1295

Table S3. The search strategy (Embase)

Search number	Query	Results
1	'amyotrophic lateral sclerosis'/exp	53856
2	'randomized controlled trial'/exp	870160
3	'als dementia':ti,ab,kw OR 'als amyotrophic lateral sclerosis':ti,ab,kw OR 'amyotrophic lateral sclerosis':ti,ab,kw OR 'amyotrophic lateral sclerosis parkinsonism dementia complex 1':ti,ab,kw OR 'amyotrophic lateral sclerosis with dementia':ti,ab,kw OR 'charcot disease':ti,ab,kw OR 'dementia with amyotrophic lateral sclerosis':ti,ab,kw OR 'gehrig disease':ti,ab,kw OR 'gehrigs disease':ti,ab,kw OR 'guam disease':ti,ab,kw OR 'guam form of amyotrophic lateral sclerosis':ti,ab,kw OR 'lou gehrig disease':ti,ab,kw OR 'lou gehrigs disease':ti,ab,kw	44917
4	'randomised controlled study':ti,ab,kw OR 'randomised controlled trial':ti,ab,kw OR 'randomized controlled study':ti,ab,kw OR 'randomized controlled trial':ti,ab,kw OR 'random*':ti,ab,kw OR 'trial, randomized controlled':ti,ab,kw OR 'clinical trials, randomized':ti,ab,kw OR 'trials, randomized clinical':ti,ab,kw	2178590
5	(#1 OR #3) AND (#2 OR #4)	2077

Table S4. The search strategy (Web of Science)

Search number	Query	Results
1	"TS=((ALS amyotrophic lateral sclerosis) OR (als dementia) OR (ALS Amyotrophic Lateral Sclerosis) OR (Amyotrophic Lateral Sclerosis) OR (Amyotrophic Lateral Sclerosis Parkinsonism Dementia Complex 1) OR (Amyotrophic Lateral Sclerosis With Dementia) OR (Charcot Disease) OR (Dementia With Amyotrophic Lateral Sclerosis) OR (Gehrig Disease) OR (Gehrigs Disease) OR (Guam Disease) OR (Guam Form of Amyotrophic Lateral Sclerosis) OR (Lou Gehrig Disease) OR (Lou Gehrigs Disease)) and Preprint Citation Index"	105205
2	"TS=((randomised controlled study) OR (randomised controlled trial) OR (randomized controlled study) OR (randomized controlled trial) OR (random*) OR (trial, randomized controlled) OR (Clinical Trials, Randomized) OR (Trials, Randomized Clinical)) and Preprint Citation Index ″	4470702
3	#1 AND #2 and Preprint Citation Index	2630
