# Supplementary material for: Efficacy and safety of pharmacological and biological therapies for amyotrophic lateral sclerosis: a network meta-analysis
Source: Front Neurol. 2026 Apr 24;17:1754716. doi: 10.3389/fneur.2026.1754716 (PMC13154608; doi:10.3389/fneur.2026.1754716)
Supplement: Supplementary file 7 [file Data_Sheet_2.doc]

**Supplementary Material 2:** Local inconsistency analysis by treatment.

(A) ALSFRS-R


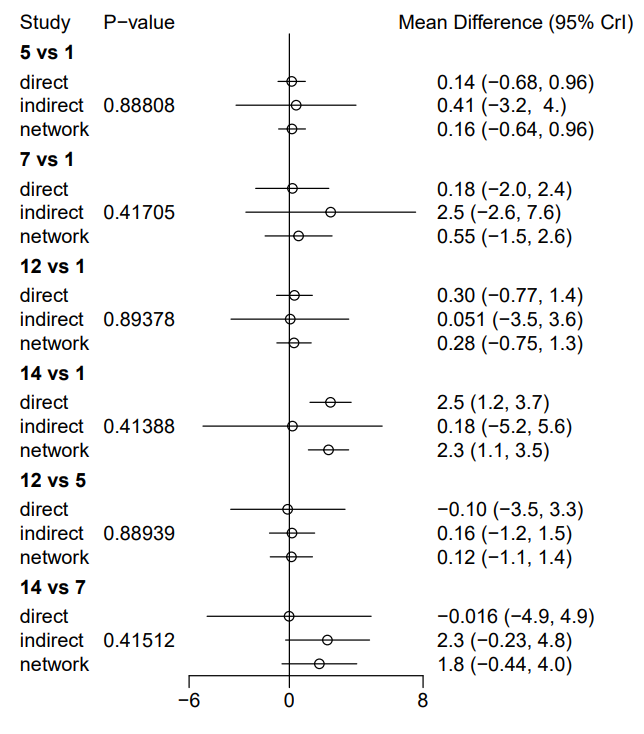


(B) Mortality rate


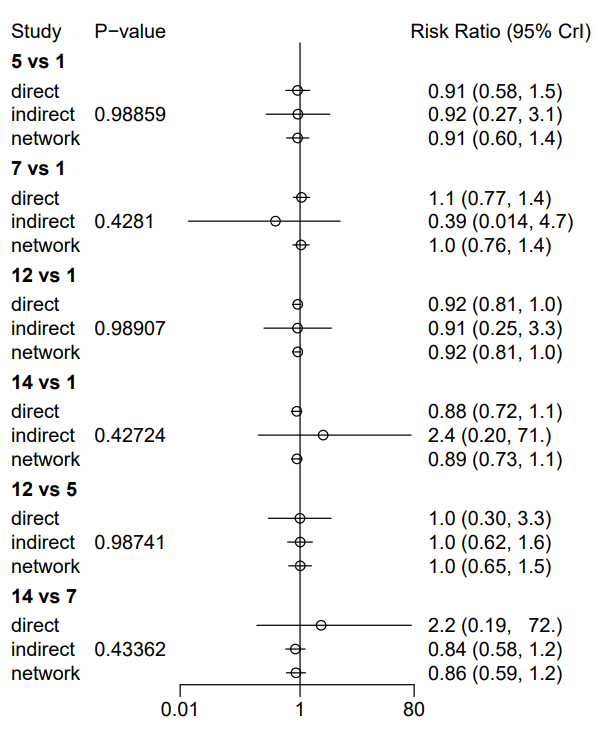


(C) SAEs


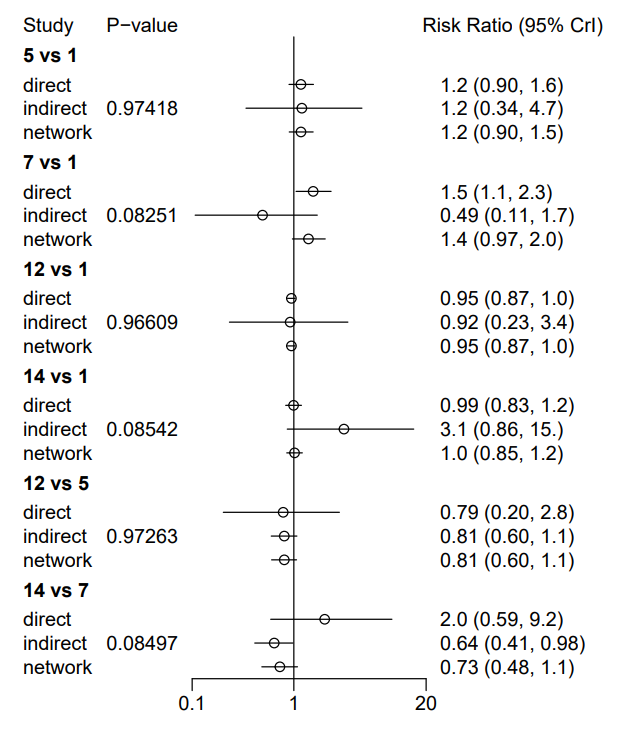


(D) Treatments ID

| id | description |
| --- | --- |
| 1 | Placebo |
| 2 | Cytokine |
| 3 | Immunosuppressant |
| 4 | Complement Inhibitor |
| 5 | Ion Channel Modulators |
| 6 | Receptor Agonist |
| 7 | Receptor Antagonist |
| 8 | Enzyme Inhibitor |
| 9 | Antioxidants |
| 10 | Cell Signaling Modulators |
| 11 | Antisense Oligonucleotide |
| 12 | Neuroprotective Agent |
| 13 | Cell Therapy |
| 14 | Nutritional Supplement |
| 15 | Alkaloid |
| 16 | Microbial Therapeutics |
| 17 | Mood Stabilizer |
| 18 | Chemically Modified Lipid Therapy |
| 19 | Nanomedicine |
| 20 | Immunomodulators |
| 21 | Chinese Herbal Medicine |
| 22 | Radiation Therapy |
| 23 | Receptor Modulator+Enzyme Inhibitor |
| 24 | Free Radical Scavenger+Neuroprotective Agent |
| 25 | Enzyme Inhibitor+Cell Signaling Modulators |
| 26 | Cell Therapy+Neuroprotective Agent |
