# Supplementary figures and images for: Efficacy and safety of pharmacological and biological therapies for amyotrophic lateral sclerosis: a network meta-analysis
Source: Front Neurol. 2026 Apr 24;17:1754716. doi: 10.3389/fneur.2026.1754716 (PMC13154608; doi:10.3389/fneur.2026.1754716)

**Supplementary Material 3:** PSRF convergence plots.

(A) ALSFRS-R


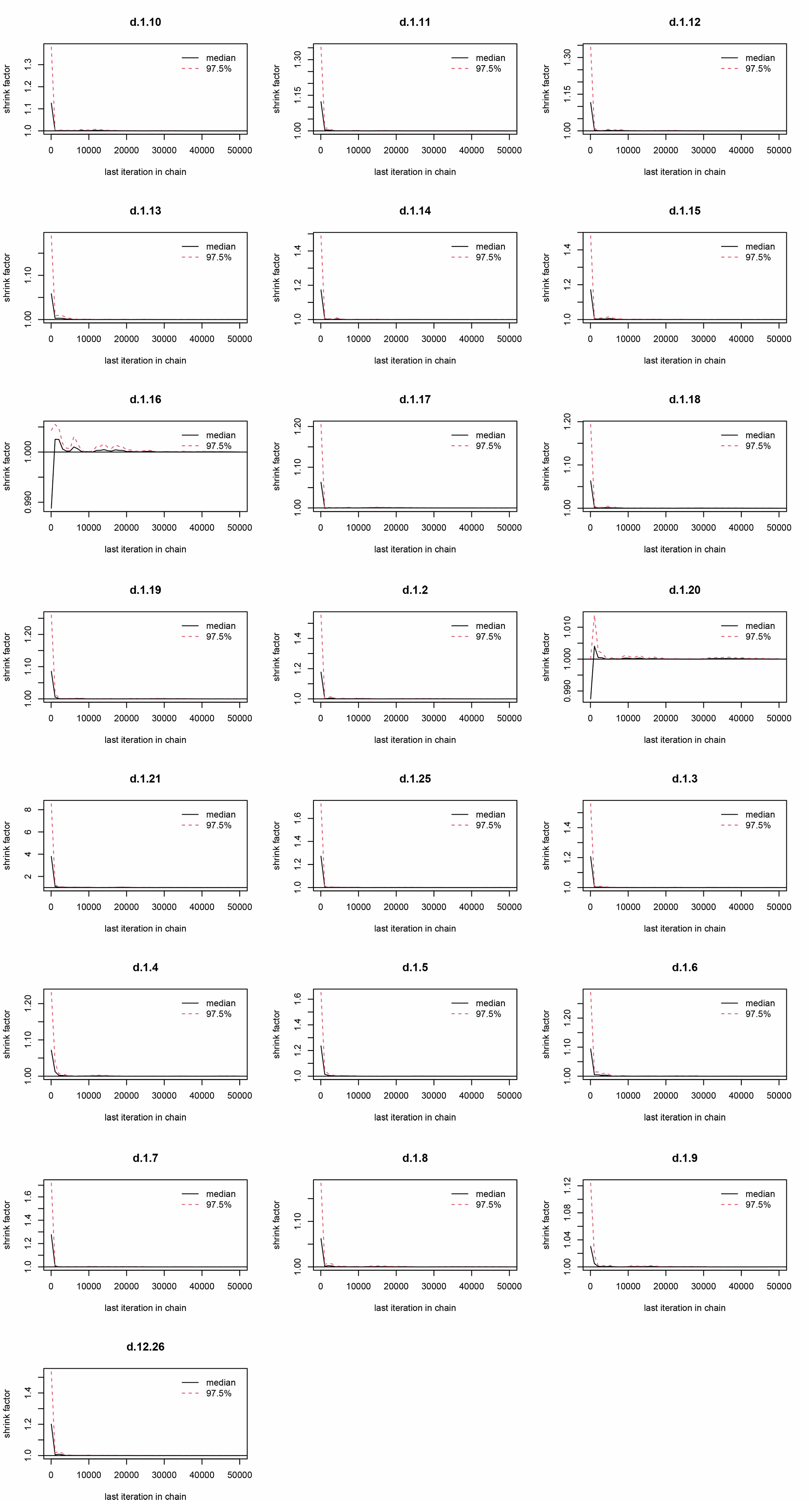


(B) FVC


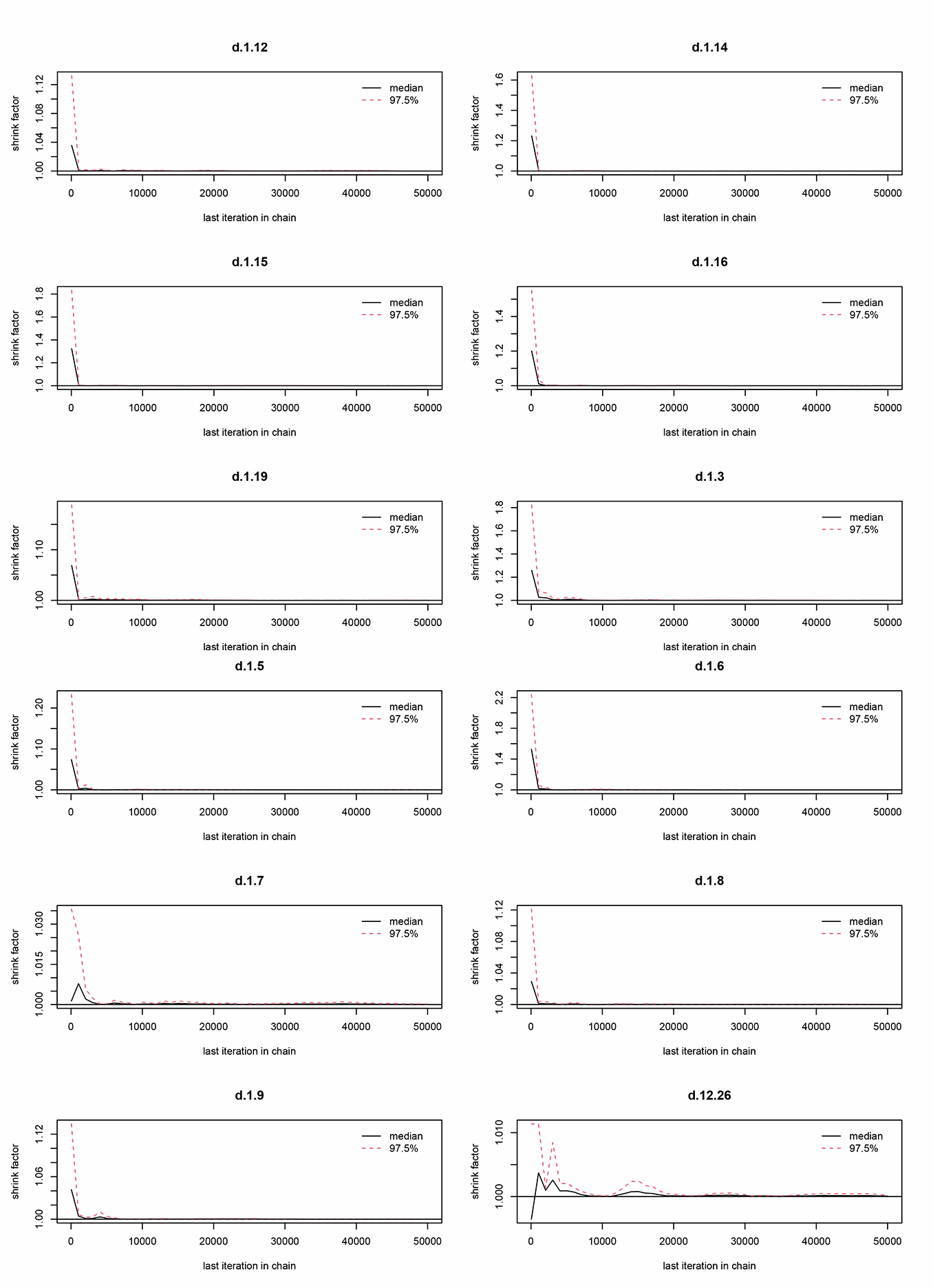


(C) Mortality rate


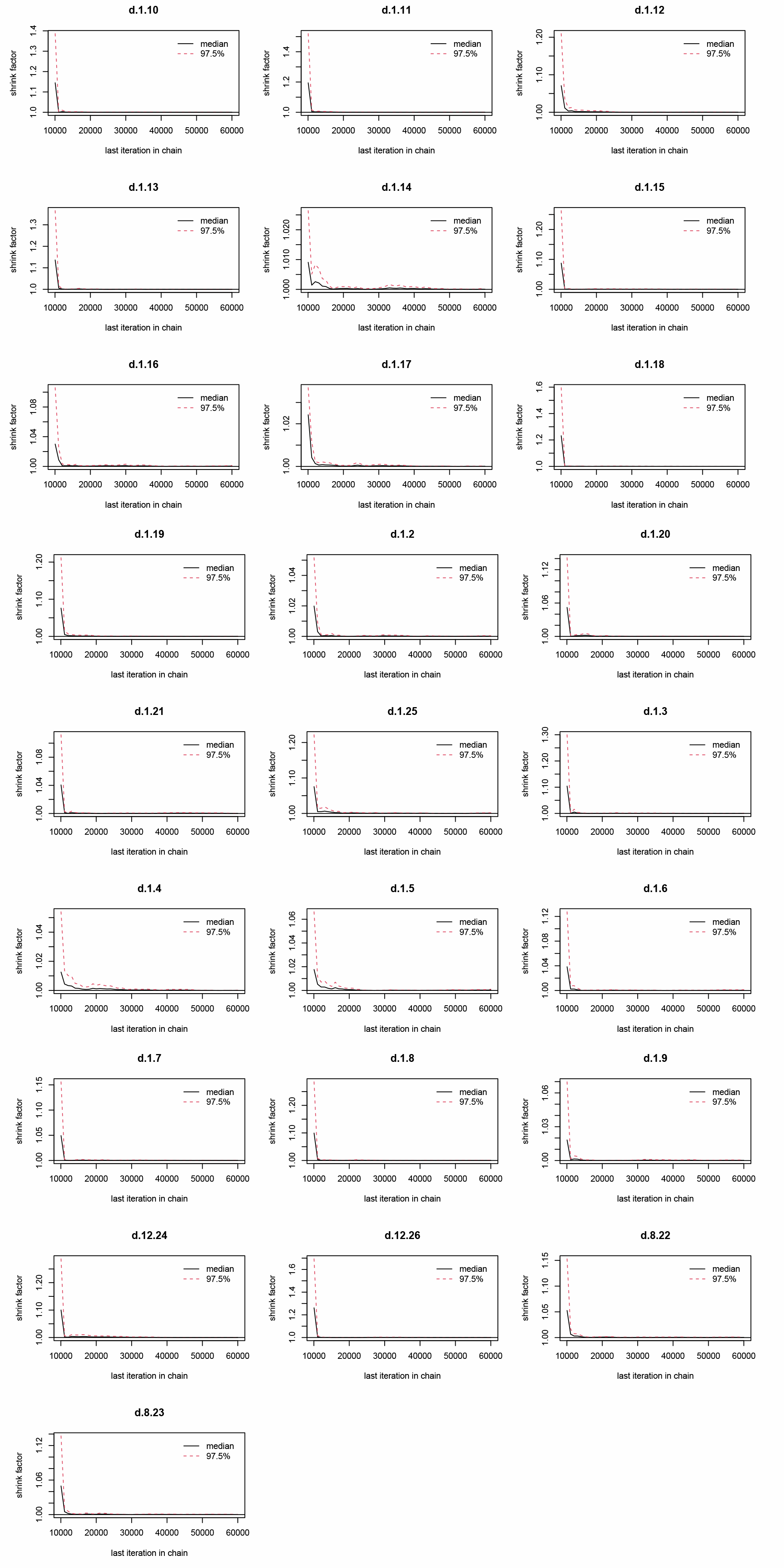


(D) SAEs


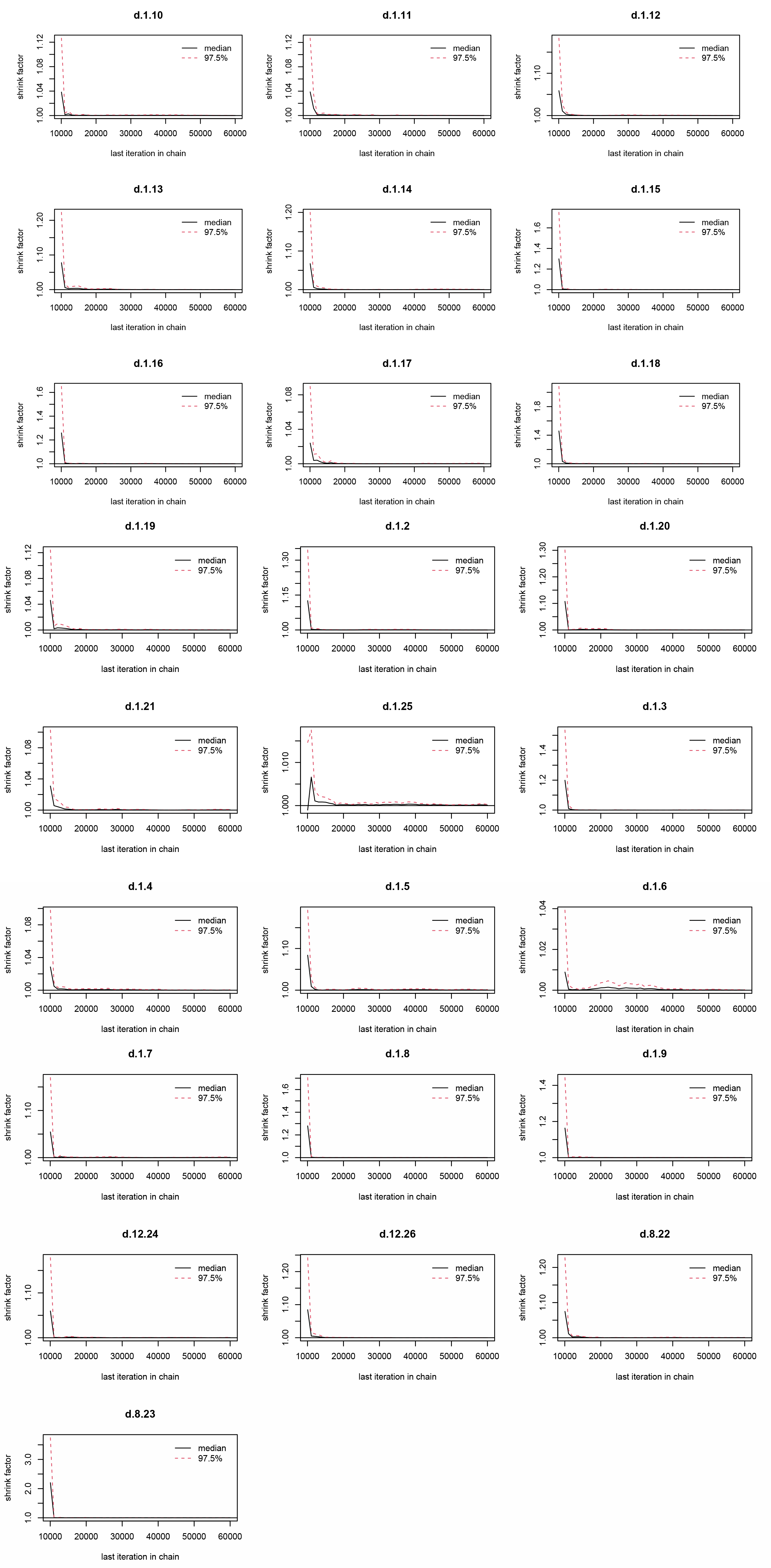

Supplement: Supplementary file 8 [file Data_Sheet_3.doc]

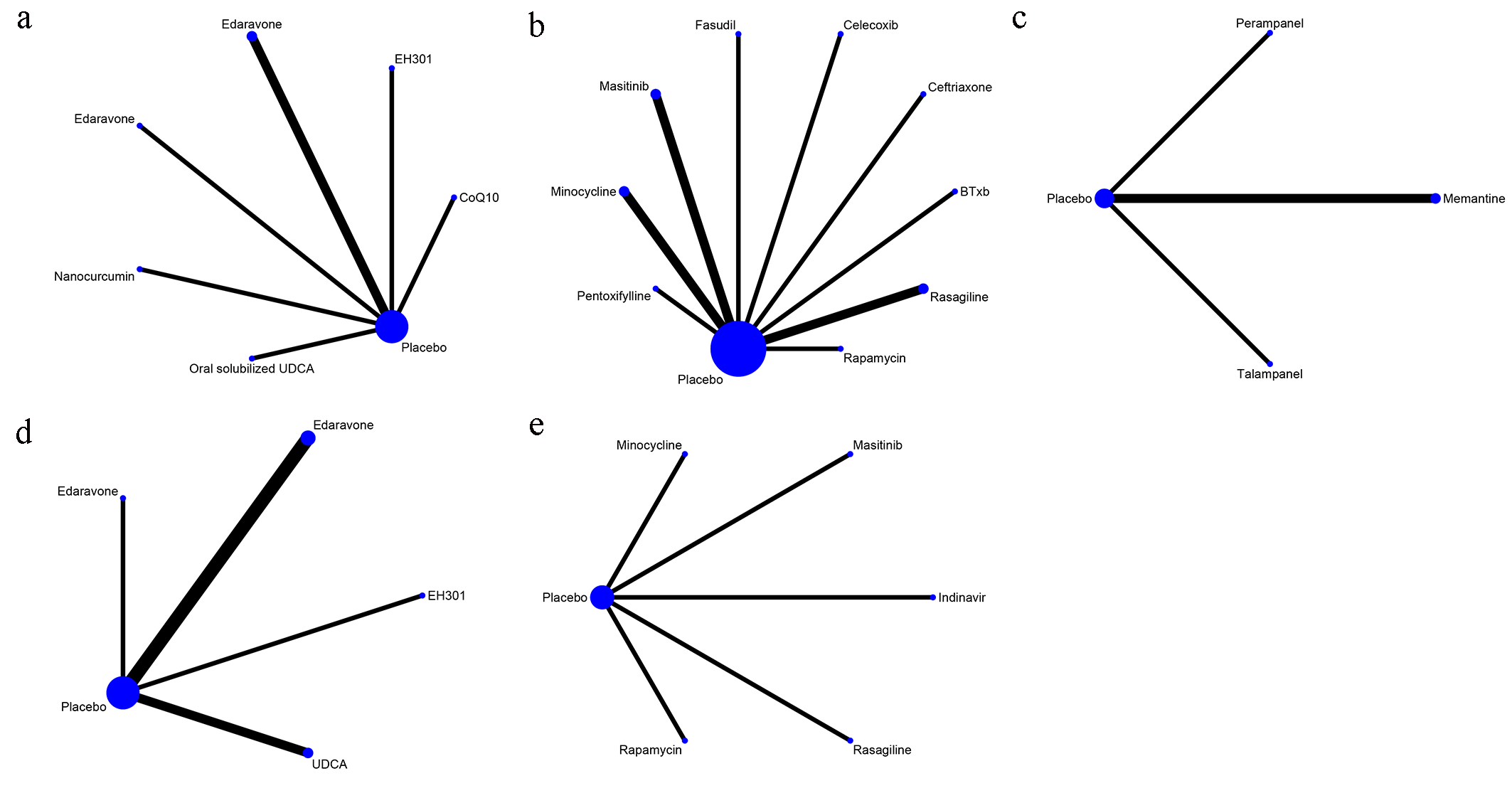

Supplement: Supplementary file 11 [file Image_1.tif]
