# Supplementary material for: Efficacy and safety of pharmacological and biological therapies for amyotrophic lateral sclerosis: a network meta-analysis
Source: Front Neurol. 2026 Apr 24;17:1754716. doi: 10.3389/fneur.2026.1754716 (PMC13154608; doi:10.3389/fneur.2026.1754716)
Supplement: Supplementary file 10 [file Data_Sheet_5.docx]

**Supplementary Material 5**

**(a) League table of mean differences (MD, 95% CrI) for ALSFRS-R Enzyme Inhibitor between interventions.**

League table of mean differences (MD, 95% CrI) for ALSFRS-R between interventions.

| Placebo |  |  |  |  |  |  |  |  |  |  |
| --- | --- | --- | --- | --- | --- | --- | --- | --- | --- | --- |
| -0.36 (-2.6, 1.89) | Fasudil |  |  |  |  |  |  |  |  |  |
| 2.81 (-3.32, 8.89) | 3.16 (-3.38, 9.65) | Rapamycin | |  |  |  |  |  |  |  |
| **3.72 (2.74, 4.69)** | **4.08 (1.62, 6.52)** | 0.91 (-5.27, 7.13) | Masitinib |  |  |  |  |  |  |  |
| 1.73 (-0.27, 3.76) | 2.09 (-0.92, 5.13) | -1.07 (-7.51, 5.39) | -1.99 (-4.22, 0.26) | Rasagiline |  |  |  |  |  |  |
| 1.08 (-0.63, 2.77) | 1.43 (-1.39, 4.23) | -1.72 (-8.08, 4.63) | -2.65 (-4.6, -0.69) | -0.66 (-3.29, 1.97) | Ceftriaxone | |  |  |  |  |
| 3.37 (-4.36, 11.2) | 3.73 (-4.35, 11.89) | 0.56 (-9.32, 10.55) | -0.35 (-8.15, 7.54) | 1.64 (-6.37, 9.74) | 2.29 (-5.64, 10.33) | BTxb |  |  |  |  |
| -2.34 (-4.08, -0.59) | -1.98 (-4.83, 0.85) | -5.14 (-11.49, 1.23) | -6.06 (-8.05, -4.06) | -4.07 (-6.75, -1.41) | -3.42 (-5.85, -0.97) | -5.71 (-13.75, 2.21) | Minocycline | |  |  |
| -0.72 (-2.48, 1.03) | -0.37 (-3.21, 2.49) | -3.53 (-9.87, 2.87) | -4.44 (-6.46, -2.43) | -2.45 (-5.13, 0.2) | -1.8 (-4.24, 0.66) | -4.09 (-12.11, 3.85) | 1.62 (-0.87, 4.09) | Pentoxifylline | |  |
| 0.01 (-2.33, 2.34) | 0.36 (-2.88, 3.62) | -2.8 (-9.32, 3.77) | -3.71 (-6.25, -1.19) | -1.72 (-4.81, 1.35) | -1.07 (-3.95, 1.83) | -3.36 (-11.52, 4.72) | 2.35 (-0.56, 5.26) | 0.73 (-2.18, 3.65) | Celecoxib |  |
| -4.14 (-6.92, -1.36) | -3.77 (-7.37, -0.23) | -6.94 (-13.66, -0.23) | -7.86 (-10.81, -4.91) | -5.88 (-9.3, -2.45) | -5.22 (-8.46, -1.95) | -7.51 (-15.82, 0.73) | -1.8 (-5.08, 1.49) | -3.42 (-6.7, -0.13) | -4.15 (-7.78, -0.52) | Minocycline |

Note: Each cell shows the MD (95% CrI) for the intervention in the row compared with that in the column. A higher MD indicates a better ALS Functional Rating Scale–Revised (ALSFRS-R) score. Bolded values denote statistically significant improvements.

| treatment | X |
| --- | --- |
| Masitinib | 0.910093 |
| BTxb | 0.751432 |
| Rapamycin | 0.73935 |
| Rasagiline | 0.714033 |
| Ceftriaxone | 0.632149 |
| Celecoxib | 0.449695 |
| Placebo | 0.443836 |
| Fasudil | 0.388638 |
| Pentoxifylline | 0.320804 |
| Minocycline | 0.124751 |
| Minocycline | 0.025221 |

**(b) League table of mean differences (MD, 95% CrI) for ALSFRS-R Antioxidants between interventions.**

League table of mean differences (MD, 95% CrI) for ALSFRS-R between interventions.

| Placebo |  |  |  |  |  |
| --- | --- | --- | --- | --- | --- |
| **3 (0.06, 5.96)** | EH301 |  |  |  |  |
| 8.53 (-0.28, 17.34) | 5.53 (-3.8, 14.87) | Nanocurcumin | |  |  |
| 1.02 (-0.98, 2.99) | -1.99 (-5.55, 1.55) | -7.52 (-16.54, 1.53) | Edaravone |  |  |
| 4.51 (-1.7, 10.73) | 1.51 (-5.38, 8.38) | -4.01 (-14.77, 6.74) | 3.5 (-3.01, 10.04) | Oral solubilized UDCA | |
| 0.64 (-1.97, 3.23) | -2.37 (-6.32, 1.55) | -7.9 (-17.11, 1.29) | -0.38 (-3.64, 2.9) | -3.88 (-10.65, 2.86) | CoQ10 |

Note: Each cell shows the MD (95% CrI) for the intervention in the row compared with that in the column. A higher MD indicates a better ALS Functional Rating Scale–Revised (ALSFRS-R) score. Bolded values denote statistically significant improvements.

| treatment | x |
| --- | --- |
| Nanocurcumin | 0.903764 |
| Oral solubilized UDCA | 0.708992 |
| EH301 | 0.635622 |
| Edaravone | 0.353262 |
| CoQ10 | 0.277713 |
| Placebo | 0.120647 |

**(c) League table of mean differences (MD, 95% CrI) for ALSFRS-R Receptor Agonist between interventions.**

League table of mean differences (MD, 95% CrI) for ALSFRS-R between interventions.

| Placebo |  |  |  |
| --- | --- | --- | --- |
| -1.39 (-4.75, 1.98) | Memantine | |  |
| -4.4 (-10.51, 1.71) | -3.01 (-9.99, 3.99) | Perampanel | |
| 2.99 (-0.3, 6.27) | 4.38 (-0.33, 9.05) | 7.38 (0.43, 14.32) | Talampanel |

Note: Each cell shows the MD (95% CrI) for the intervention in the row compared with that in the column. A higher MD indicates a better ALS Functional Rating Scale–Revised (ALSFRS-R) score. Bolded values denote statistically significant improvements.

| treatment | x |
| --- | --- |
| Talampanel | 0.969988 |
| Placebo | 0.58293 |
| Memantine | 0.34859 |
| Perampanel | 0.098492 |

**(d) League table of mean differences (MD, 95% CrI) for FVC Enzyme Inhibitor between interventions.**

League table of mean differences (MDs, 95% CrI) in FVC between interventions.

| Placebo |  |  |  |  |  |
| --- | --- | --- | --- | --- | --- |
| -7.88 (-33.72, 18.13) | Rapamycin | |  |  |  |
| **7.5 (4.69, 10.32)** | 15.38 (-10.72, 41.4) | Masitinib |  |  |  |
| 2.99 (-6.43, 12.27) | 10.81 (-16.83, 38.42) | -4.52 (-14.34, 5.16) | Rasagiline |  |  |
| -1.98 (-5.07, 1.09) | 5.89 (-20.22, 31.94) | -9.49 (-13.67, -5.3) | -4.97 (-14.74, 4.96) | Minocycline | |
| -10.54 (-24.67, 3.54) | -2.67 (-32.12, 26.82) | -18.05 (-32.45, -3.71) | -13.49 (-30.35, 3.49) | -8.55 (-23.06, 5.87) | Indinavir |

Note: Each cell shows the mean difference (MD) with 95% credible intervals (CrIs) for the intervention in the row compared with that in the column. A higher MD indicates a higher forced vital capacity (FVC), while 0 indicates no difference between the two interventions. Bolded values represent statistically significant increases in FVC.

| treatment | x |
| --- | --- |
| Masitinib | 0.938385 |
| Rasagiline | 0.694841 |
| Placebo | 0.56353 |
| Minocycline | 0.362804 |
| Rapamycin | 0.302582 |
| Indinavir | 0.137858 |

**(e) League table of mean differences (MD, 95% CrI) for FVC Antioxidants between interventions.**

League table of mean differences (MDs, 95% CrI) in FVC between interventions.

| Placebo |  |  |  |
| --- | --- | --- | --- |
| **16.39 (7.22, 25.41)** | EH301 |  |  |
| 1.04 (-2.63, 4.72) | -15.35 (-25.11, -5.46) | Edaravone |  |
| **7.34 (2.86, 11.83)** | -9.03 (-19.1, 1.15) | 6.3 (0.51, 12.13) | UDCA |

Note: Each cell shows the mean difference (MD) with 95% credible intervals (CrIs) for the intervention in the row compared with that in the column. A higher MD indicates a higher forced vital capacity (FVC), while 0 indicates no difference between the two interventions. Bolded values represent statistically significant increases in FVC.

|  | x |
| --- | --- |
| EH301 | 0.98618 |
| UDCA | 0.674305 |
| Edaravone | 0.243193 |
| Placebo | 0.096322 |
